# Fungal Diversity and Community Composition of Culturable Fungi in *Stanhopea trigrina* Cast Gibberellin Producers

**DOI:** 10.3389/fmicb.2018.00612

**Published:** 2018-04-04

**Authors:** Sonia Salazar-Cerezo, Nancy Martinez-Montiel, Maria del Carmen Cruz-Lopez, Rebeca D. Martinez-Contreras

**Affiliations:** ^1^Laboratorio de Ecología Molecular Microbiana, Centro de Investigaciones en Ciencias Microbiológicas, Instituto de Ciencias, Benemérita Universidad Autónoma de Puebla, Puebla, Mexico; ^2^Centro de Investigación en Biotecnología Aplicada, Instituto Politécnico Nacional, Tepetitla, Mexico

**Keywords:** fungal diversity, orchid, *Stanhopea tigrina*, endemic, endophytes, epiphytes, gibberellins

## Abstract

*Stanhopea tigrina* is a Mexican endemic orchid reported as a threatened species. The naturally occurring microorganisms present in *S. tigrina* are unknown. In this work, we analyzed the diversity of endophytic and epiphytic culturable fungi in *S. tigrina* according to morphological and molecular identification. Using this combined approach, in this study we retrieved a total of 634 fungal isolates that presented filamentous growth, which were grouped in 134 morphotypes that were associated to 63 genera, showing that *S. tigrina* harbors a rich diversity of both endophytic and epiphytic fungi. Among these, the majority of the isolates corresponded to Ascomycetes, with *Trichoderma* and *Penicillium* as the most frequent genera followed by *Fusarium* and *Aspergillus*. Non-ascomycetes isolated were associated only to the genus *Mucor* (Mucoromycota) and *Schizophyllum* (Basidiomycota). Identified genera showed a differential distribution considering their epiphytic or endophytic origin, the tissue from which they were isolated, and the ability of the orchid to grow on different substrates. To our knowledge, this work constitutes the first study of the mycobiome of *S. tigrina*. Interestingly, 21 fungal isolates showed the ability to produce gibberellins. Almost half of the isolates were related to the gibberellin-producer genus *Penicillium* based on morphological and molecular identification. However, the rest of the isolates were related to the following genera, which have not been reported as gibberellin producers so far: *Bionectria, Macrophoma, Nectria, Neopestalotiopsis, Talaromyces, Trichoderma*, and *Diplodia*. Taken together, we found that *S. tigrina* possess a significant fungal diversity that could be a rich source of fungal metabolites with the potential to develop biotechnological approaches oriented to revert the threatened state of this orchid in the near future.

## Introduction

Fungi play a central role in most ecosystems and they have important functions in soil and plant habitats. It is now clear that the microbiota associated with plants contribute to maintain their biological diversity in terrestrial ecosystems through different biological processes (Coats and Rumpho, 2014; Hardoim et al., 2015). In this regard, the fungal population associated with plants contributes to the adaptation process in response to biotic and abiotic stress, it is involved in the defense against pathogens and it has been related to the production of secondary metabolites with antimicrobial activity or with the synthesis of different phyto-hormones including gibberellins (Tsavkelova et al., 2008; Hajiboland et al., 2010; Contreras-Cornejo et al., 2011; Khan et al., 2012, 2015b). Gibberellins play an essential role in regulation of growth and development of angiospermic plants (Bahalla et al., 2010), they are associated with several plant growth and development processes as seed germination, stem elongation, flowering, and fruit development (Bilkay et al., 2010; Lu et al., 2016).

Fungi can be found as epiphytes (on the surface of the plant) or as endophytes (inside the plant tissue). Both populations are growing apart only by millimeters nevertheless they differ greatly in their composition (Santamaría and Bayman, 2005). Epiphytes grow on the surface with contact to outer environment, whereas endophytes are in contact with the inner microenvironment. It is unclear to what extent plants control which epiphytes are able to enter the leaf, and to what extent endophytes may affect this process and the mycelial growth of fungi complicates the difference between both pupulations, while comparison of endophytic and epiphytic floras may help to determine the basis for selectivity. The coexistence of epiphytic and endophytic microorganisms contributes to maintain the conditions needed for plant health, plant protection (Andrews and Harris, 2000; Santamaría and Bayman, 2005; Rodriguez et al., 2009), and microbial biodiversity (Huang et al., 2008; Kharwar et al., 2010), allowing a global homeostatic state. For this reason, further knowledge of the fungal mycobiota residing in plants is decisive to understand fungal ecology and to generate approaches for plant conservation (Yuan et al., 2009).

Mexico possess a great diversity of orchids with around 1,260 species and 170 genera (Hágsater et al., 2005; Soto et al., 2007) from which 40% correspond to endemic species. Unfortunately, in spite of being one of the largest families of flowering plants of the planet, it is also of the most threatened. Different factors including pollution, climate change, and human activities (Rands et al., 2010) have had a negative impact on orchid diversity. In Mexico, 188 orchid species are categorized as endangered by the government (SEMARNAT, 2010). *Stanhopea* is a neotropical orchid genus consisting of approximately 40 species out of which 13 occur in Mexico. Although the species belonging to this genus are widespread through Mexico, Central, and South America, some of the species are endemic in Mexico. *Stanhopea tigrina* is an endemic orchid in Mexico that is considered threatened at the state-level. This orchid shows beautiful massive flowers (18 cm in diameter) with a sweet and penetrating aroma that makes them very attractive for marketing (Soto-Arenas and Solano-Gómez, 2007) and due to its Mexican endemic nature, it was chosen as the symbol of the Mexican Orchid Association. Concerns about the decline of *S. tigrina* populations in Puebla State and in Mexico require the development of efficient and ecologically viable strategies to preserve the species. Given the importance of the microbiome in the extension of the plants' ability to adapt to many kinds of environmental conditions and changes (Vandenkoornhuyse et al., 2015), it is important to identify the associated microbiome in order to develop strategies that allow its conservation. On the other hand, fungi isolated from orchids may serve as beneficial species that could be used in orchid propagation and reintroduction, improving acclimatization and vigor (Brundrett, 2007; Yuan et al., 2009). However, to our knowledge there are no reports dealing with the diversity of fungi living in the *S. tigrina*, one of the most spectacular orchids in Mexico.

In this work, we analyzed the fungal diversity of both epiphytic and endophytic fungi in the threatened Mexican endemic orchid *S. tigrina* (collected from Cuetzalan del Progreso, Puebla, México) according to morphological and molecular identification. Additionally, we examined the ability of the isolates to produce gibberellins and we uncovered several fungal genera as gibberellin producers. Our results indicate that *S. tigrina* harbors a rich diversity for both epiphytic and endophytic fungi that could be a source of metabolites with a positive effect on the development of this endangered species. With this work, we want to contribute to the knowledge of the filamentous fungal diversity associated to *S. tigrina* but also to provide some tools that could allow the development of alternative strategies that could help to revert the threatened state of this orchid.

## Materials and methods

### Study site

Samples were taken from the Botanical Garden Xoxoctic (20°2′22.63″N, 97°30′32.16″W) located in the municipality of Cuetzalan del Progreso, Puebla, Mexico, which corresponds to a natural area devoted to preserve and investigate the biological richness of the Sierra Norte de Puebla. This location corresponds to a tropical mountain cloud forest ecosystem, to an altitude of 1000 masl, with semi-warm to warm humid climate and rainfall throughout the year (PIGEU, 2009). Considering the endangered status for this species, only six plants and one flower of *S. tigrina* were collected, from which one was lithophyte, two grew as epiphytes, and three were cultivated on a substrate that contained coconut fiber and soil.

### Fungal isolation and cultivation

For fungal isolation, we took 2 g of fresh and healthy tissue for each part of the plant (leaf, pseudobulb, root, or flower). The procedure was performed for the six plants independently. To isolate fungal epiphytes, weighed and washed in 100 ml of isotonic saline solution with constant stirring for 3 min. Then, tissues were removed and with the washing suspension we performed serial dilutions (10^−1^, 10^−2^, and 10^−3^) and 100 μl of each dilution were plated in triplicates in different media: Potato Dextrose Agar (PDA, MCD LAB), Malt Extract Agar medium (MEA, Difco), and V8 juice agar. The composition of these media is the following; PDA: potato infusion 20%, dextrose 2%, agar 1.5%; MEA: malt extract 1.2%, dextrin 0.2%, glycerol 02%, peptone 0.07%, agar 1.5%; V8 juice agar: V8 juice 200 ml/l, 3 g/l CaCO_3_, and 20 g/l agar. To recover fungal endophytes, tissues were rinsed with water and surface-sterilized in a sequence of 70% ethanol for 2 min followed by 1% NaClO_2_ during 2 min, and finally rinsed twice in sterile distilled water. Disinfected plant tissues were cut and macerated. The extract obtained was recovered and serial dilutions of 10^−1^, 10^−2^, and 10^−3^ were performed. 100 μl of each dilution was plated on PDA, MEA, and V8 juice agar; the plating was performed in triplicate. Rhizospheric fungi were also isolated by taking 1 g of sample which was placed in 100 ml of isotonic saline and the resulting suspension was used to perform serial dilutions of 10^−1^, 10^−2^, and 10^−3^; from each dilution, 100 μl were taken and plated on PDA, MEA, and V8 juice agar. All plates were incubated at 28°C for 1 month. Plates were checked daily and each emerging fungal colony was transferred onto a new PDA plate until axenic cultures were obtained.

### Phenotypic identification of the fungal isolates

Individual strains with apparent distinct morphology were sub-cultured until obtaining a pure isolate. In order to identify distinct and representative morphologies, isolates were plated on PDA and incubated for 1 week at 28°C. To induce sporulation or formation of distinctive structures that could be used to identify fungal genus, isolates were grown under microculture conditions. Briefly, a sterile PDA agar cube (1 cm) was cut and placed on a slide where the fungal isolate was inoculated and then a coverslip was placed on top. This system was deposited in a petri dish with humid chamber environment and incubated for 1 week at 28°C. After the incubation, the coverslip was removed and the fungal growth was stained with lactophenol cotton blue. Morphological characteristics of the isolates were challenged by the identification keys. These isolates were then classified into morphotypes according to the growth rate, color, texture, and morphology of their colonies. Non-sporulating fungal morphotypes were grown in different media such as malt extract agar (MEA), Czapek agar (CZ), oat agar (OA) among others, combined with different light periods to stimulate sporulation.

### Molecular identification of the fungal morphotypes

To further analyze the identity of the fungal isolates recovered, different samples of each morphotype were considered according to the number of members in each group. When the morphotype included 1–3 elements, we used only one isolate to perform the molecular identification. In the same fashion, we used 3 isolates for groups with 7–10 elements and 5 isolates for groups with more than 10 elements. For these fungi, DNA was extracted as previously reported (Liu et al., 2000). Fresh mycelium was scraped from the surface of the agar plate and transferred into a clean tube with 500 μl of lysis buffer (400 mM Tris-HCl [pH 8.0], 60 mM EDTA [pH 8.0], 150 mM NaCl, 1% sodium dodecyl sulfate) and the mixture was vortexed in the presence of glass beads. After 30 min, 150 μl of precipitation solution (3 M potassium acetate, 5 M acetate, pH 4.8) were added to the tube, vortexed briefly and centrifuged at 10,000 rpm for 5 min. The supernatant was recovered and centrifuged again. Precipitation was performed with an equal volume of isopropyl alcohol at −80°C for 20 min with centrifugation at 10,000 rpm for 5 min. DNA pellet was washed with 300 μl of 70% ethanol. After centrifugation at 10,000 rpm for 5 min, the supernatant was discarded and the DNA pellet was dissolved in 40 μl of water.

Fungal ITS-region and partial LSU were amplified using primers ITS1F (Gardes and Bruns, 1993) and LR5 (Hopple and Vilgalys, 1994; Moncalvo et al., 2000), which are specific for fungi. The resulting PCR products ranged from 1.3 to 1.7 kb in size. The LSU region is useful to identify fungi in public databases. PCR reactions were performed with 2 μl of genomic DNA in a total volume of 25 μl, using Taq DNA polymerase with ThermoPol buffer, according to the manufacturer's instructions (New England BioLabs) and further purified with the High Pure PCR purification kit (Roche). The following thermocycling program was used: 95°C for 5 min (1 cycle); 94°C for 35 s, 55°C 35 s, 72°C for 2 min (35 cycles); and 72°C for 10 min (1 cycle). Amplification products were sequenced using primers ITS1F and LR5 using the services provided by Macrogen (Korea). Two sequencing rounds were performed in order to confirm the genetic features. Sequences obtained in this study were annotated in the GenBank (Supplementary Material).

### Sequence analysis

Forward and reverse sequence reads were analyzed using the CLC genomics workbench (QIAGEN). BLAST searches (www.ncbi.nlm.nih.gov/BLAST) were conducted to determine the close known relative for each case. Isolates were assigned as closest to a certain genus when identity was ≥95% (Sánchez et al., 2008; Chen et al., 2012; Tan et al., 2012). The evolutionary history was inferred by using the Maximum Likelihood Phylogeny 1.2 method with the Jukes Cantor nucleotide substitution model. The analysis involved 45 nucleotide sequences with a total of 100 replicates in the bootstrap analysis. Evolutionary analyses were conducted in CLC Main Workbench 7.9.1.

### Qualitative determination of gibberellins

Pure fungal isolates were incubated in 5 ml of ICI medium, a medium with a low concentration of nitrogen that allows the production of gibberellins, for 10 days at 30°C under dark conditions and agitation (200 rpm). After incubation, 0.2 ml of culture media were recovered and combined with 0.2 ml of ethanol (96%, v/v) and 2 ml of a cold mixture of sulfuric acid/ethanol (1:1 v/v). This mixture was incubated at 48°C for 30 min (Candau et al., 1992) and then exposed to an UV lamp. Samples showing green fluorescence were considered positive for gibberellin production.

### Quantitative determination of gibberellins by HPLC

Seven-day old mycelium of each strain was inoculated in a flask with 100 ml of Czapek broth (1% glucose, 1% peptone) and incubated at 30°C in the presence of light (as described by Bahalla et al., 2010). After 10 days, the culture was filtered and 50 ml of the filtrate was recovered and the pH adjusted to 3 by adding HCl (1N). The extraction was performed three times using ethyl acetate (1:1). The organic phase was separated and passed through anhydrous sodium sulfate. The solvent was eliminated in a rotary evaporator at 40°C and 10 rpm. The residue was dissolved in 50 μl of HPLC grade methanol and used for reverse phase HPLC analysis. The analysis was done in triplicate in a Hewlett Packard HPLC instrument (series 1100) with operation and data analysis using Chemstation software running on a HP Vectra 486 computer (Hewlett Packard Co.). Gibberellins were chromatographed using LiChrospher on a RP-18 column (250 × 4 mm i.d.). Acetonitrile and acidic water (0.01% H_3_PO_4_*)* in a 70:30 ratio was used as mobile phase with a flow rate of 0.6 ml/min for 15 min. One microliter of each sample was injected and gibberellins were identified at 206 nm (Barendse et al., 1980; Bahalla et al., 2010). Gibberellin quantification was performed as previously reported (Bahalla et al., 2010). Full spectra were obtained for commercial GA_3_ and GA_4_ (1 μg/μl, Sigma) that were used as standard.

## Results

**Figure 1:**
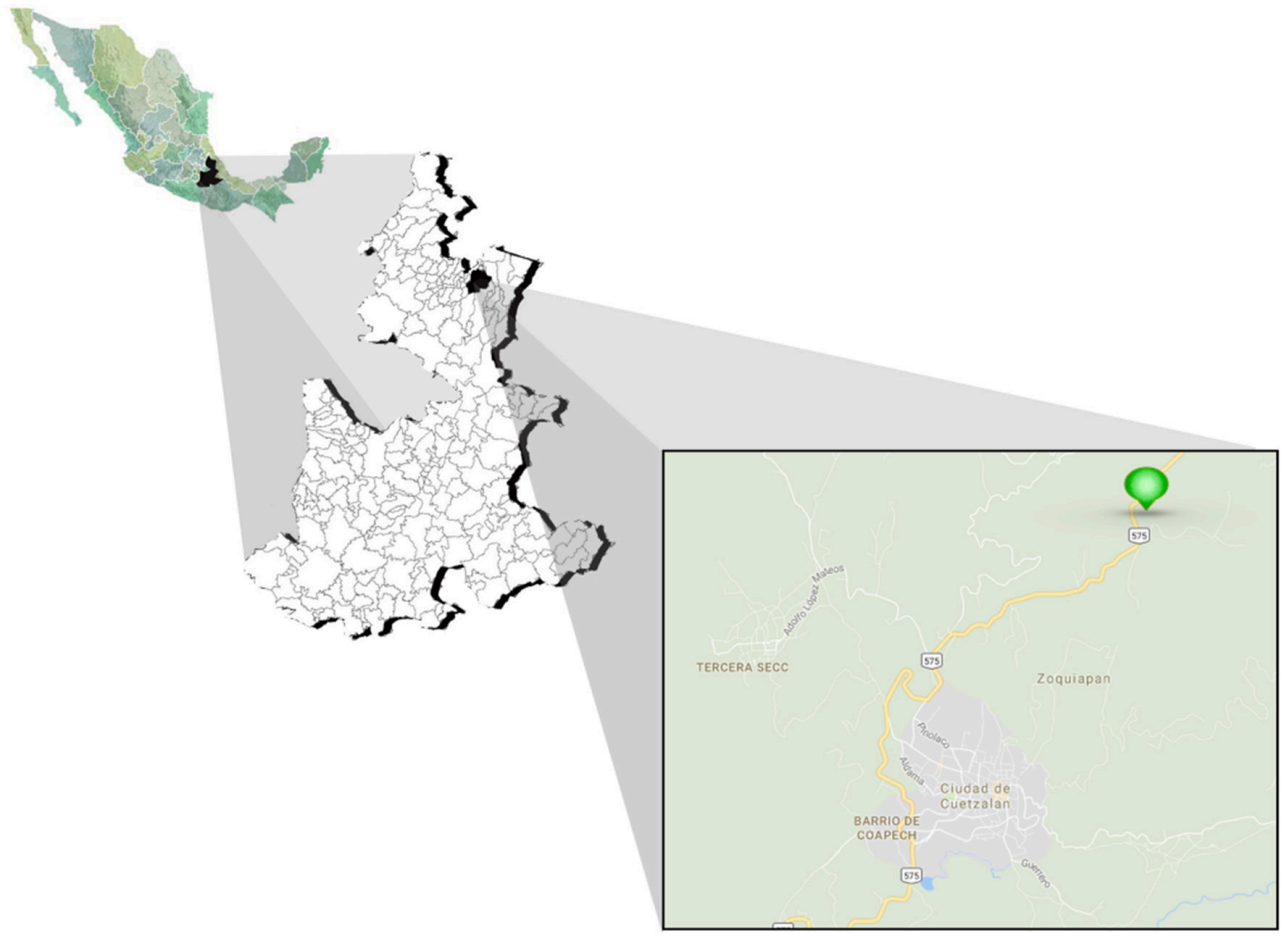
Location map of the study site. Sampling was performed in the Botanical Garden Xoxoctic (Cuetzalan del Progreso, Puebla, México), a natural area located to the Sierra Norte (Puebla) devoted to preserve species characteristic of this region.

In this work, a total of 891 isolates were recovered from six plants of *S. tigrina* collected from the botanical garden Xoxoctic in Puebla, Mexico (Figure 1). Out of the 891 fungal isolates recovered from the six *S. tigrina* plants, 257 isolates did not show filamentous characteristics, but were confirmed as yeast according to Gram and Lactophenol Cotton Blue staining. Moreover, their ability to produce gibberellins was also tested but any of the yeast showed the ability to produce this metabolite under the conditions assayed (data not shown).

For the 634 fungal isolates that presented filamentous growth, morphological identification was carried out according to colony and hyphal morphology of the macroscopic culture and characteristics of the spores under microculture conditions. According to these characteristics, the 634 fungal isolates were classified into 139 distinct morphotypes, according to the morphological characteristics observed (Supplementary Table 1). Insights into the distribution and diversity of the fungal isolates recovered from *S. tigrina* is further presented.

### Distribution of fungal isolates in *S. tigrina*

In addition to the morphological characterization, molecular analyses were carried out to confirm the identity of the different morphogroups (Wang et al., 2005; Jin et al., 2013). Using this combinatory approach, we identified 134 morphotypes that comprised 63 genera, while 10 isolates grouped in 5 morphotypes could not be identified.

**Figure 2:**
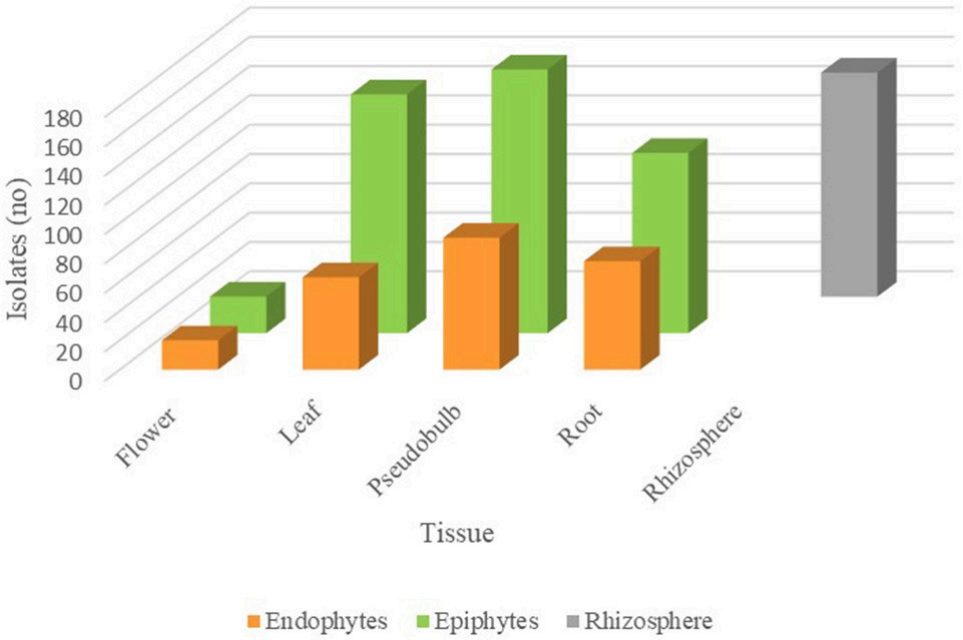
Abundance of fungal isolates in different *S. tigrina* tissues. The number of endophytic (orange), epiphytic (green), and rhizospheric (gray) fungal isolates recovered from different tissues is shown.

From the total culturable population recovered, 80% of the isolates were epiphytes, while 20% of them were endophytes. Considering tissue distribution, the greatest number of isolates were recovered from pseudobulb (270 isolates), followed by the leaf and root with 226 and 197 isolates, respectively. A total of 153 isolates were found in the rhizosphere and 45 in the flower (Figure 2). In general, the proportion of epiphytes than endophytes isolated was similar for the different tissues studied. Surprisingly, the flower resulted a very rich tissue if we keep in mind that we only recovered one flower during the sampling process. Considering that the flowers of this orchids only last a few days, the appearance of the flower at the sampling time was timely.

**Figure 3:**
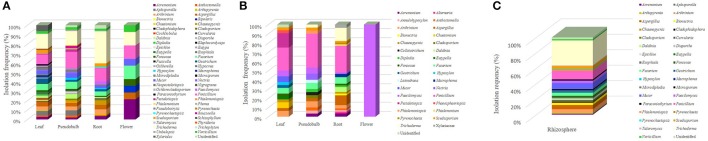
Tissue distribution of the fungal genera isolated from *S. tigrina*. Epiphytic **(A)** and endophytic **(B)** isolates were recovered from the leaf, pseudobulb, root, and flower of *S. tigrina* and the relative frequency for the different genera isolated is shown. Genera recovered from the rhizosphere are also shown **(C)**.

Concerning the genera identified, a total of 63 genera was established, being *Trichoderma* the most recurrent group identified showing 124 isolates, while the second most frequently recovered was *Penicillium* with 103 isolates (Figure 3). These were the dominant genera retrieved from *S. tigrina*, followed by *Fusarium* and *Aspergillus* with 40 and 33 isolates, respectively. The infrequent groups that presented only one isolate included *Alternaria, Cladophialophora, Cochliobolus, Colletotrichum, Fusicolla, Gibberella, Hypocrea, Phialemonium, Phoma, Pseudobotrytis, Schizophyllum*, and *Talaromyces*. Considering the provenance of the isolate, we found that the epiphytic population comprised 57 genera, while 32 genera constituted the endophytic population. On the other hand, we identified 33 genera in the rhizospheric community (Figure 3). Even when a total of 25 genera were found both in the endophytic and epiphytic populations, we also noticed differences in the composition of the two groups. In this regard, 32 genera were found only in the epiphytic population, while the following fungal taxa were recovered only as endophytes: *Alternaria, Annulohypoxylon, Anthostomella, Colletotrichum, Letendraea*, and *Phaeosphaeriopsis* (Figure 3). Interestingly, *Trichoderma* was frequently isolated both as epiphyte and from the rhizosphere.

**Figure 4:**
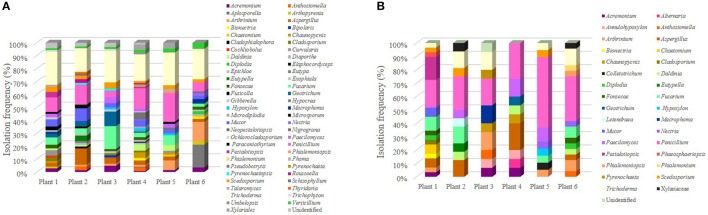
Distribution of culturable fungi in different *S. tigrina* plants. The relative isolation frequency was estimated for the epiphytic **(A)** and endophytic **(B)** isolates that were recovered from each of the six different plants. Plants 1 and 2 grew as epiphytes, plant 3 was growing as litophyte, while plants 4, 5, and 6 were growing on substratum.

We next analyzed the genera distribution for the different plants (Figure 4) and we found that plant 4, which was grown on substratum showed the highest number of associated epiphytic genera, while plant three that grew as litophyte presented the lowest number of fungal genera isolated (Figure 4A). Concerning the endophytic community, plant 1 showed the greatest diversity of genera (Figure 4B).

### Tissue distribution of the fungal isolates

In this study, we recovered fungal isolates from the following tissues in *S. tigrina*: leaf, pseudobulb, root, and flower. In the roots, the presence of mycorrhizal fungi was sought, however, the presence of pelotons was not observed and no isolates belonging to this type of fungi was obtained. The composition and abundance of the isolated genera varied according to the host tissues investigated. The tissue with the greatest number of associated genera was the leaf with 40, followed by pseudobulb with 32 and 27 from the root, while only 10 isolates were recovered from the flower (Figure 2). For all the tissues studied, epiphytes were retrieved from the surface, while endophytes were obtained from macerated disinfected tissue. Fungal isolates were also recovered from the associated rhizosphere. Out of the 45 genera recovered from the leaf, 12 were found in both the endophytic and epiphytic populations, while 30 genera were recovered only as epiphytes. In the case of the pseudobulb, we found 11 genera in both populations, whereas 19 genera were associated to the epiphyte population and five genera were exclusively endophytic. In the root, 12 genera were ubiquitous while 10 and 8 genera were found only as epiphytic or endophytic, respectively. Finally, in the flower we identified 9 epiphytic genera and only *Paecilomyces* was encountered as endophyte (**Table 2**).

Regarding tissue distribution of epiphytes, the leaf was the tissue with the greatest diversity with 38 genera associated, followed 29 in pseudobulb, 21 in root and only 9 in flower, while 33 genera were recovered from the rhizosphere (Figure 3). The most frequent fungal genera were *Trichoderma, Penicillium, Fusarium, Mucor*, and *Aspergillus*, which were found in all tissues (Figure 3A). However, we found tissue specificity for some genera. This was the case for *Cochliobolus, Diplodia, Elaphocordyceps, Eutypa, Fusicolla, Gibberella, Hypocrea, Nigrograna, Phoma, Thyridaria* that were recovered only from the leaf. On the other hand, *Phialemonium, Pseudobotrytis, Roussoella*, and *Umbelopsis* were found only in pseudobulb while *Bionectria, Cladophialophora, Microsporum, Phialemoniopsis, Pyrenochaeta*, and *Talaromyces* seemed exclusive from rhizosphere.

On the other hand, for the endophytic population we found a more balanced abundance of fungal isolates. In this case, out of the 32 genera identified, 20 were found in the root, 16 in pseudobulb, 15 in leaves and only one in flower (Table 1). Regarding the identity of the isolated genera, only *Paecilomyces* was identified in all tissues. Consistent with our observations of the epiphytic population, we discovered the following tissue-specificity: *Chaetomium, Eutypella, Geotrichum, Hypoxylon, Mucor, Pestalotiopsis, Phaeosphaeriopsis* were identified only in the leaf, *Alternaria, Bionectria, Daldinia, Macrophoma, Phialemonium*, and *Pyrenochaeta* were found only in the root, while *Cladosporium, Colletotrichum, Letendraea, Nectria*, and *Phialemoniopsis* were retrieved only from the pseudobulb (Figure 3B).

**Table 1:** Tissue distribution for the different fungal isolates recovered from *S. tigrina*.

	**Leaf**		**Pseudobulb**		**Root**		**Flower**		**Rhizosphere**	**Total**	
**Genus**	**Endo**	**Epi**	**Endo**	**Epi**	**Endo**	**Epi**	**Endo**	**Epi**		**Endo**	**Epi**
*Acremonium*	0	4	1	3	2	3	0	3	1	3	14
*Alternaria*	0	0	0	0	1	0	0	0	0	1	0
*Annulohypoxylon*	0	0	1	0	1	0	0	0	0	2	0
*Anthostomella*	0	2	1	3	1	1	0	0	0	2	6
*Aplosporella*	0	7	0	8	0	0	0	0	1	0	16
*Arthopyrenia*	0	1	0	0	0	1	0	0	1	0	3
*Arthrinium*	2	6	2	6	2	4	0	0	6	6	22
*Aspergillus*	1	7	1	6	6	4	0	1	7	8	25
*Bionectria*	0	0	0	0	1	0	0	0	3	1	3
*Bipolaris*	0	1	0	0	0	0	0	1	0	0	2
*Chaetomium*	2	2	0	1	0	0	0	0	0	2	3
*Chaunopycnis*	1	0	0	3	1	4	0	0	1	2	8
*Cladophialophora*	0	0	0	0	0	0	0	0	1	0	1
*Cladosporium*	0	1	1	2	0	1	0	0	1	1	5
*Cochliobolus*	0	1	0	0	0	0	0	0	0	0	1
*Colletotrichum*	0	0	1	0	0	0	0	0	0	1	0
*Curvularia*	0	1	0	0	0	1	0	0	2	0	4
*Daldinia*	0	3	0	7	3	1	0	0	1	3	12
*Diaporthe*	0	1	0	2	0	0	0	0	1	0	4
*Diplodia*	0	2	1	0	1	0	0	0	0	2	2
*Elaphocordyceps*	0	1	0	0	0	0	0	0	0	0	1
*Epichloe*	0	0	0	2	0	0	0	0	1	0	3
*Eutypa*	0	1	0	0	0	0	0	0	0	0	1
*Eutypella*	1	2	0	0	0	0	0	0	1	1	3
*Exophiala*	0	0	0	1	0	0	0	0	1	0	2
*Fonsecae*	1	2	0	3	1	1	0	1	2	2	9
*Fusarium*	2	10	2	11	4	4	0	2	5	8	32
*Fusicolla*	0	1	0	0	0	0	0	0	0	0	1
*Geotrichum*	1	7	0	3	0	2	0	0	1	1	13
*Gibberella*	0	1	0	0	0	0	0	0	0	0	1
*Hypocrea*	0	1	0	0	0	0	0	0	0	0	1
*Hypoxylon*	1	0	0	3	0	1	0	0	1	1	5
*Letendraea*	0	0	1	0	0	0	0	0	0	1	0
*Macrophoma*	0	3	0	0	2	0	0	0	1	2	4
*Microdiplodia*	0	2	0	0	0	0	0	0	1	0	3
*Microsporum*	0	0	0	0	0	0	0	0	2	0	2
*Mucor*	2	5	0	7	0	2	0	1	10	2	25
*Nectria*	0	1	2	2	0	0	0	0	0	2	3
*Neopestalotiopsis*	0	4	0	4	0	1	0	0	0	0	9
*Nigrograna*	0	1	0	0	0	0	0	0	0	0	1
*Ochlorocladosporium*	0	1	0	1	0	0	0	0	0	0	2
*Paecilomyces*	2	1	2	1	1	3	1	0	4	6	9
*Paraconiothyrium*	0	2	0	0	0	0	0	0	2	0	4
*Penicillium*	8	15	11	25	16	12	0	2	14	35	68
*Pestalotiopsis*	5	1	0	4	0	0	0	0	0	5	5
*Phaeosphaeriopsis*	1	0	0	0	0	0	0	0	0	1	0
*Phialemoniopsis*	0	0	1	0	0	0	0	0	1	1	1
*Phialemonium*	0	0	0	1	1	0	0	0	0	1	1
*Phoma*	0	1	0	0	0	0	0	0	0	0	1
*Pseudobotrytis*	0	0	0	1	0	0	0	0	0	0	1
*Pyrenochaeta*	0	0	0	0	1	0	0	0	1	1	1
*Pyrenochaetopsis*	0	0	0	0	0	1	0	0	1	0	2
*Roussoella*	0	0	0	3	0	0	0	0	0	0	3
*Scedosporium*	1	5	1	1	1	3	0	0	5	3	14
*Schizophyllum*	0	1	0	0	0	0	0	0	0	0	1
*Talaromyces*	0	0	0	0	0	0	0	0	1	0	1
*Thyridaria*	0	1	0	0	0	0	0	0	0	0	1
*Trichoderma*	0	22	1	22	8	28	0	2	41	9	115
*Trichophyton*	0	1	0	0	0	1	0	0	0	0	2
*Umbelopsis*	0	0	0	2	0	0	0	0	0	0	2
*Verticillium*	0	4	0	4	0	2	0	1	2	0	13
*Xylariaceae*	0	0	0	0	2	0	0	0	0	2	0
*Xylariales*	0	9	0	0	0	0	0	0	0	0	9
Unidentified	1	0	0	3	0	3	0	0	3	1	9

*Endo, Endophytes; Epi, Epiphytes*.

### Fungal distribution according to *S. tigrina* growth habit

**Figure 5:**
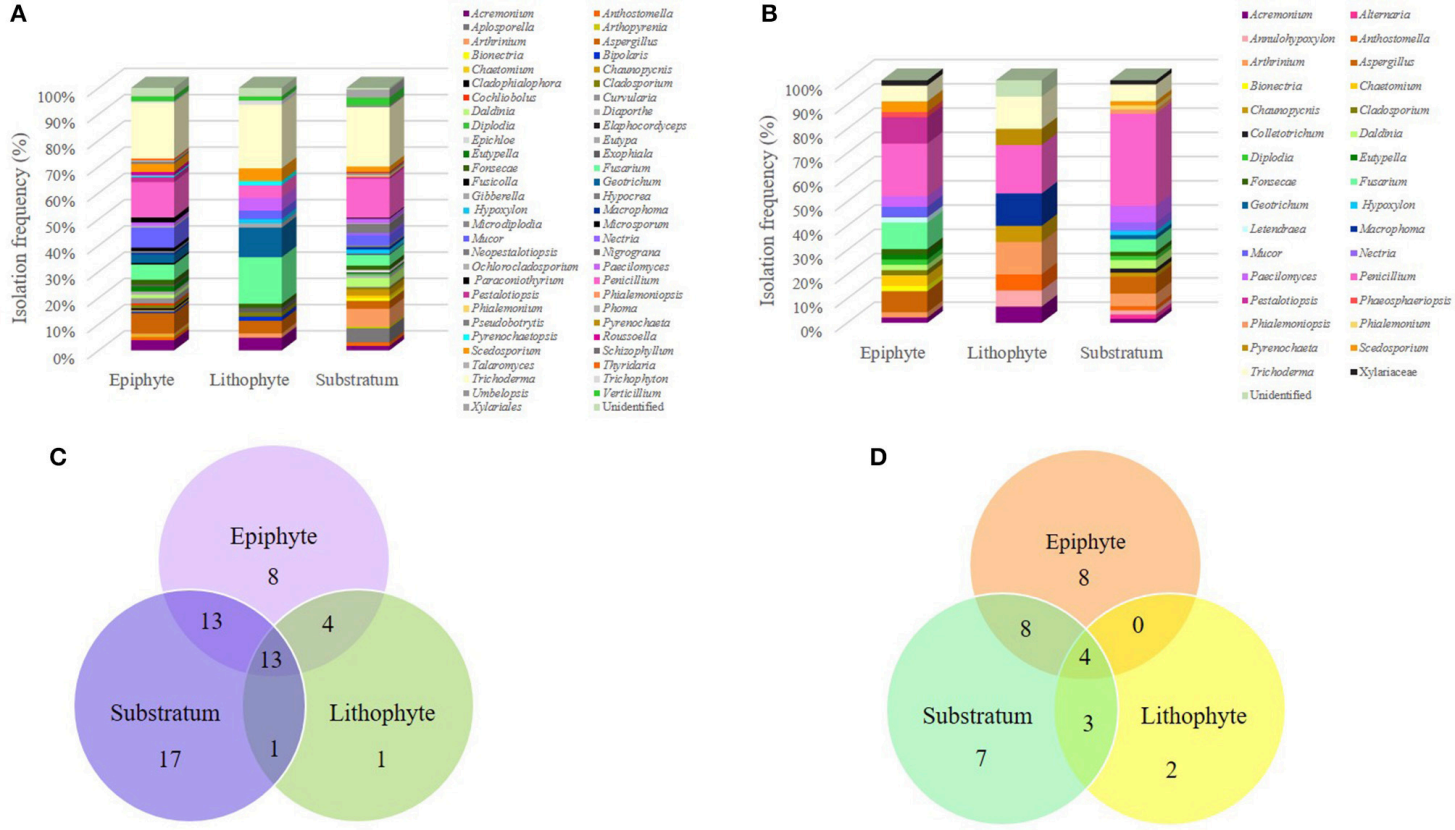
Fungal community composition according to the different growth habits of *S. tigrina*. Isolation frequency of genera growing as epiphytes, as litophytes, or in substratum are presented for the epiphytic **(A)** and endophytic **(B)** fungal communities. The number of genera shared by plants with different growth habits is shown for the epiphytic **(C)** and endophytic **(D)** fungal communities.

Considering the ability of *S. tigrina* to grow in different substrates, in this study we recovered fungal isolates from one lithophyte plant, two epiphyte specimens and three orchids that grew on substrate. When we compared the abundance of genera according to these growth conditions, we realized that there were 65 genera recovered from plants that grew as epiphytes and 58 from orchids grown on substrate. The similar abundance of genera observed in orchids grown as epiphytes or on the substrate was noticed for both the epiphytic and endophytic fungal populations, albeit their composition was different. On the other hand, we recovered only 28 genera from the lithophyte orchid. Insights into the special composition of the epiphytic and endophytic fungal populations discovered are further presented (Figure 5).

Considering the endophytic fungal population, all shared a core of four genera: *Acremonium, Arthrinium, Penicillium*, and *Trichoderma*. The genera shared between the epiphyte orchids and those grown on substrate were *Aspergillus, Daldina, Diplodia, Fonsecae, Fusarium, Paecilomyces*, and *Scedosporium*. Orchids grown on substrate shared the genera *Annulohypoxylon, Anthostomella*, and *Chaunopycnis* with the lithophyte plant, while the epiphyte and lithophyte specimens did not show any common genus. In addition, some genera seemed to be exclusive from a particular environment (Table 2). This was the case for *Macrophoma* and *Pyrenochaeta* present only in the litophyte plant. Regarding the epiphytic fungal population, all orchids shared a core of 13 genera: *Acremonium, Arthrinium, Aspergillus, Cladosporium, Fonsecae, Fusarium, Geotrichum, Mucor, Paecilomyces, Penicillium, Scedosporium, Trichoderma*, and *Verticillium*. It was also observed that the substrate and epiphyte plants shared 12 genera: *Anthostomella, Arthopyrenia, Chaunopycnis Curvularia, Daldinia, Diaporthe, Macrophoma, Microdiplodia, Ochlorocladosporium, Paraconiothyrium Pestalotiopsis*, and *Roussoella*. Epiphytic and litophyte plants shared the genus *Bipolaris, Exophiala, Trichophyton*, and *Pyrenochaetopsis*. Besides, in the substrate plants were found *Aplosporella, Bionectria, Chaetomium, Diplodia, Elaphocordyceps, Epichloe, Hypocrea, Nectria, Neopestalotiopsis, Nigrograna, Phialemoniopsis, Phoma, Pseudobotrytis, Pyrenochaeta*, and *Umbelopsis* as non-shared genera, we found nine genera associated only with epiphytic plants, and *Giberella* in lithophytes (Table 2). On the other hand, *Eutypella* was found only in epiphyte habit plants, both in the endophytic and epiphytic populations whereas *Nectria, Phialemoniopsis*, and *Phialemonium* were found only in plants grown on substrate.

**Table 2:** Fungal distribution in the different *S. tigrina* plants according to its growth habit.

**Core (Plants)**	**Epiphyte vs. Lithophyte (Plants)**	**Lithophyte vs. substrate plants**	**Epiphyte vs. substrate plants**	**Epiphyte (Plants)**	**Lithophyte (Plants)**	**Substrate (Plants)**
**ENDOPHYTES GENERA**						
*Acremonium* *Arthrinium* *Penicillium* *Trichoderma*		*Annulohypoxylon* *Anthostomella* *Chaunopycnis*	*Aspergillus* *Daldina* *Diplodia* *Fonsecae* *Fusarium* *Paecilomyces* *Scedosporium* *Xylariaceae*	*Bionectria Chaetomium Cladosporium Eutypella Letendraea Mucor Pestalotiopsis Phaeosphaeriopsis*	*Macrophoma Pyrenochaeta*	*Alternaria* *Colletotrichum* *Geotrichum* *Hypoxylon* *Nectria* *Phialemoniopsis* Phialemonium
**EPIPHYTE GENERA**						
*Acremonium Arthrinium* *Aspergillus* *Cladosporium* *Fonsecae* *Fusarium* *Geotrichum* *Mucor* *Paecilomyces* *Penicillium Scedosporium* *Trichoderma* *Verticillium*	*Bipolaris* *Exophiala* *Pyrenochaetopsis Trichophyton*	*Hypoxylon*	*Anthostomella* *Arthopyrenia* *Chaunopycnis* *Curvularia* *Daldinia* *Diaporthe* *Macrophom* *Microdiplodia* *Ochlorocladosporium* *Paraconiothyrium Pestalotiopsis* *Roussoella*	*Cladophialophora* *Cochliobolus Eutypa* *Eutypella* *Fusicolla* *Microsporum* *Schizophyllum Talaromyces* *Thyridaria*	*Gibberella*	*Aplosporella* *Bionectria* *Chaetomium* *Diplodia* *Elaphocordyceps* *Epichloe* *Hypocrea* *Nectria* *Neopestalotiopsis* *Nigrograna* *Phialemoniopsis* *Phialemonium* *Phoma* *Pseudobotrytis* *Pyrenochaeta* *Umbelopsis* *Xylariales*

### Capability of some fungal isolates to produce gibberellins

As part of our ongoing efforts toward finding novel alternatives to revert the threatened state of *S. tigrina*, we investigated the ability of these fungal isolates to produce secondary metabolites. It is also important to identify these potentially bioactive fungi for a better understanding of the characteristics of the relevant fungal community associated to the orchid. In the search of fungal isolates from *S. tigrina* that could have a positive impact on plant development, we performed a general screening to identify gibberellin-producing isolates using a qualitative method described previously (Candau et al., 1992). Using this approach, we tested all the 891 isolates and found that 21 of them were positive for gibberellin production using the qualitative test. For these 21 isolates, the production of gibberellins was quantitatively determined by HPLC, using commercial GA_3_ (Sigma G7645) and GA_4_ (Sigma G7276) as standards (Supplementary Material). This analysis showed the presence of GA_3_ in the 21 isolates, being STF062 the isolate with the highest production of GA_3_ (4.1 μg), followed by STF624 that produced 2.0 μg of GA_3_. Other gibberellin producers were isolates STF282, STF524, STF526, STF538, STF599, STF637, STF657, STF756, and STF757, all with gibberellin production around 1 μg (Table 3). Even when the assay was designed to identify both GA_3_ and GA_4_, in most isolates only the production of GA_3_ was detected and the production of both gibberellins was observed only for isolate STF723, with a predominant production of GA_4_ (2.1 μg) over GA_3_ (0.1 μg).

**Table 3:** GA_3_ and GA_4_ production in fungal isolates determined by HPLC.

**Isolate ID**	**GA**_**3**_		**GA**_**4**_	
	**Rt (min)**	**Total content (μg)**	**Rt (min)**	**Total content (μg)**
Standard	2.763	1,000	4.047	1,000
STF062	2.745	4.1		
STF147	2.750	0.8		
STF149	2.743	0.5		
STF282	2.743	1.1		
STF343	2.733	0.6		
STF524	2.775	1.3		
STF526	2.780	1.5		
STF538	2.788	1.2		
STF591	2.731	0.6		
STF599	2.742	1.4		
STF624	2.772	2.0		
STF654	2.727	0.6		
STF664	2.720	0.5		
STF673	2.751	1.6		
STF723	2.764	0.1	4.025	2.1
STF753	2.781	0.7		
STF756	2.760	1.0		
STF757	2.783	1.2		
STF859	2.737	0.4		
STF860	2.488	0.3		
STF864	2.718	0.4		

*Rt, retention time*.

### Molecular identification of gibberellin producing isolates

In addition to the morphological characterization, molecular analyses were carried out to confirm the identity of the 21 isolates recovered from *S. tigrina* that were determined as gibberellin producers and the results are included in Table 4. The ITS1-5.8S-ITS2 sequences of these isolates were compared to the sequences annotated in the GenBank and using this approach, we found that isolates STF62, STF147, STF149, STF538, STF591, STF599, STF654, STF723, and STF859 were associated to various reported sequences for the genus *Penicillium*. Isolates STF664, STF673, and STF860 showed the highest similarity with the *Xylariales* order. The ITS sequence for the isolate STF526 did not show significant homology to any known cultured fungal species. The other isolates were associated to the following fungal species: *Arthrinium marii* (STF757)*, Bionectria cf Ochroleuca* (STF624)*, Macrophoma* (STF282)*, Nectria pseudotrichia* (STF753), *Neopestalotiopsis* sp. (STF524)*, Talaromyces funiculosus* (STF343)*, Trichoderma* sp. (STF864), and *Diplodia quercivora* (STF756).

**Table 4:** Gibberellin producing fungal isolates and their closest relatives from GenBank.

**Fungal isolate**	**GenBank accesion no**.	**Closest relative**	**Identity (%)**
UAPSTF062	MG971306	*Penicillium oxalicum*	100
UAPSTF149	MG971307	*Penicillium citrinum*	100
UAPSTF282	MG971308	*Macrophoma theicola*	100
UAPSTF343	MG971309	*Talaromyces funiculosus*	99
UAPSTF524	MG971310	*Neopestalotiopsis* sp.	99
UAPSTF538	MG971311	*Penicillium funiculosum*	99
UAPSTF591	MG971312	*Penicillium oxalicum*	100
UAPSTF599	MG971313	*Penicillium oxalicum*	100
UAPSTF624	MG971314	*Bionectria ochroleuca*	99
UAPSTF654	MG971315	*Penicillium oxalicum*	100
UAPSTF664	MG971316	*Xylaria* sp.	99
UAPSTF673	MG971317	*Xylaria* sp.	99
UAPSTF723	MG971318	*Penicillium* sp.	99
UAPSTF753	MG971319	*Nectria pseudotrichia*	100
UAPSTF756	MG971320	*Diplodia quercivora*	98
UAPSTF757	MG971321	*Arthrinium sacchari*	94
UAPSTF859	MG971322	*Penicillium oxalicum*	100
UAPSTF860	MG971323	*Xylariaceae* sp.	90
UAPSTF864	MG971324	*Trichoderma asperellum*	99

**Figure 6:**
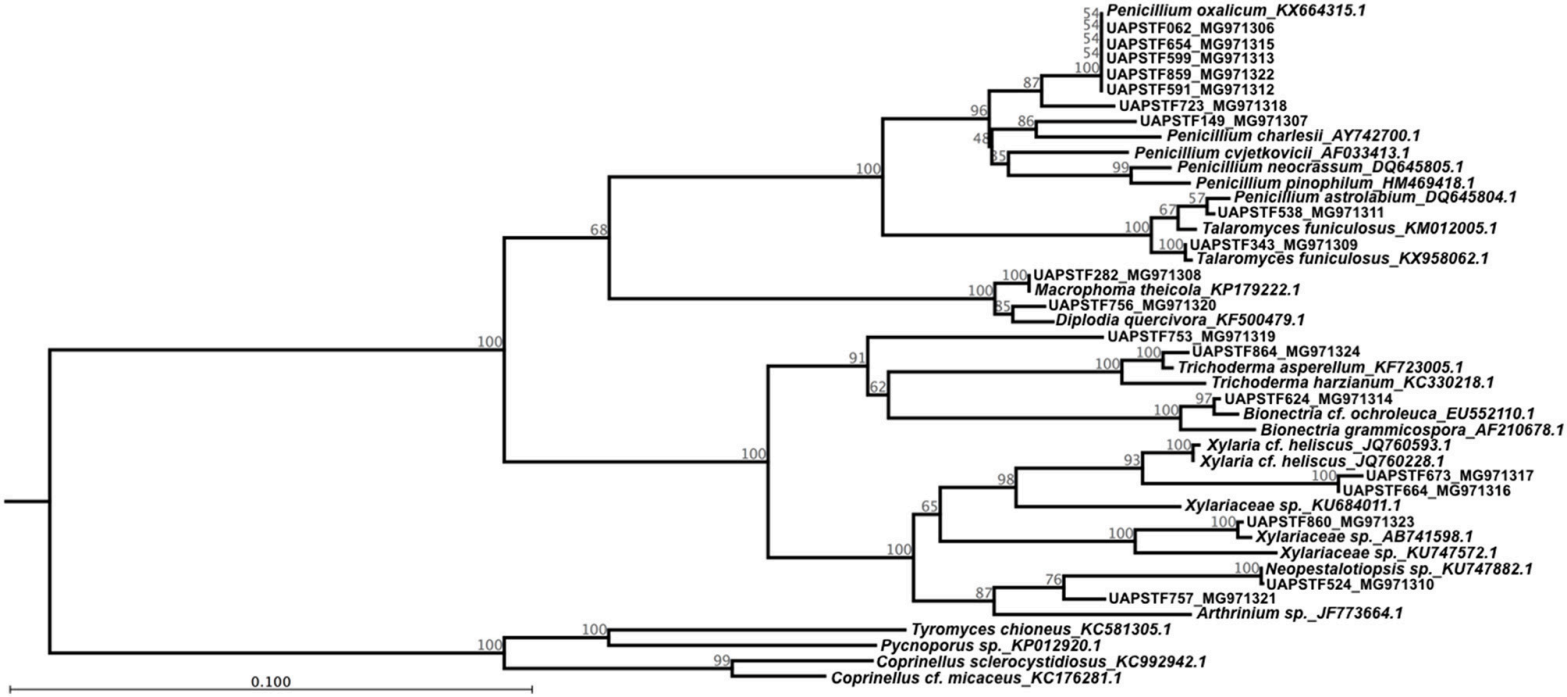
Phylogenetic relationship among the 21 fungal isolates that showed the ability to produce gibberellin. The tree with the highest log likelihood (−8844.44) is shown. The tree is drawn to scale, with branch lengths measured in the number of substitutions per site. The analysis involved 45 nucleotide sequences with a total of 100 replicates in the bootstrap analysis.

The sequences used for the molecular identification were handled in order to construct the phylogenetic tree shown in Figure 6. The phylogenetic analysis of the sequences divided the strains into two main clades. The first clade comprising 12 isolates includes two classes: Eurotiales and Botryosphaeriales. The Eurotiales clade contains several well-supported branches and represents basically two genera: *Penicillium* and *Talaromyces*. The largest group shows a bootstrap support of 96% and includes different known strains for the genus *Penicillium*. Isolates STF062, STF654, STF599, STF859, and STF599 were all grouped with *Penicillium oxalicum* and in a different branch STF723 was also associated to this group. Isolate STF149 and *Penicillium charlesii* formed a group with a bootstrap of 86%. In a different clade, isolate STF538 was grouped with *Penicillium pinophilum*, while isolate STF343 clustered together with *Talaromyces funiculosus* with a bootstrap support of 100%. Phylogenetic analysis suggests that isolates STF062, STF149, STF538, STF591, STF599, STF654, STF723, and STF859 belong to the genus *Fusarium*, while STF343 belong to the genus *Talaromyces*. The Botryosphaeriales clade was supported for a bootstrap of 100% and groups *Macrophoma theicola* with the isolate STF282 in one clade and isolate STF756 with *Diplodia quercivora* in a separate clade (Figure 6), associating the isolates with the corresponding genus.

Within the Xylariales clade, STF864 formed a group with *Trichoderma asperellum* and STF624 clustered together with *Bionectria cf. ochroleuca*, both with a bootstrap support of 100%. STF664 and STF673 were grouped with *Xylaria cf. heliscus* with a bootstrap value of 93%, while STF860 was grouped within the *Xylariaceae* family. Therefore, phylogenetic analysis suggests that STF864 belong to the genus *Trichoderma*, STF624 relates to the genus *Bionectria*, and STF664, STF673 match the genus *Xylaria*, while STF860 associates with the *Xylariaceae* family.

The final clade groups two isolates and two genera corresponding to Hypocreales. On one hand, we have isolate STF524 grouped with *Neopestalotiopsis* sp. with a strong bootstrap of 100% and in separate clades STF757 releted to *Arthrinium* sp. with a bootstrap value of 87%. Given that all the isolates identified as gibberellin producers, four sequences for reported basidiomycetes were used as outgroup.

## Discussion

### Culturable fungal diversity in *S. tigrina*

In this study we identified 63 genera, 30 of which have already been reported as orchid endophytes (McCormick et al., 2004; Albores et al., 2005; Chen et al., 2011; Jiang et al., 2011; Sudheep and Sridhar, 2012; Sawmya et al., 2013; Tao et al., 2013). Some of these endophytes have shown beneficial effects, like the antibacterial activity of *Alternaria* sp. and *Fusarium oxysporum* recovered from Brazilian orchids (Vaz et al., 2009) or the ability of *Aspergillus flavus, A. ochraceus*, and *Paecilomyces* sp. to protect the orchids *Bulbophyllum neilgherrense* and *Vanda testacea* from herbivores (Sudheep and Sridhar, 2012).

Interestingly, we report here for the first time several genera as fungal partners for orchids, including the following: *Aplosporella, Microsporum, Mucor, Trichophyton, Diplodia, Fonsecae*, and *Scedosporium*, indicating that the composition of the fungal communities associated to different orchid species should be further analyzed. Even when these genera correspond to endophytes, pathogens, or latent pathogens in other hosts (Meletiadis et al., 2002; Becker, 2003; Burgess et al., 2004; Prusky et al., 2004; Cortez et al., 2008; Lazzizera et al., 2008; Leelasuphakul et al., 2008; Slippers et al., 2009; Taylor et al., 2009; Twizeyimana et al., 2013; Dreaden et al., 2014; Dou et al., 2017), their biotechnological potential as secondary metabolite producers (Malonek et al., 2005; Bi et al., 2013) should also be considered when designing future applications to rescue this endangered orchid. In this regard, fungal isolates reported here could have a strong impact on the health of *S. tigrina* by maintaining a balance in the composition of the associated microbiome (Omacini et al., 2001), serving as a defense against different pathogens or helping to deal with biotic stress (Brundrett, 2006). Thus, a better knowledge of the fungal community associated with *S. tigrina* could help to preserve endangered species like this orchid from further destruction.

### Epiphytic and endophytic fungal communities

In this work, a total of 891 fungal isolates was recovered using a culture-dependent approach to identify both epiphytes and endophytes, revealing the richness of the fungal community present in *S. tigrina* plants. This is the first report about culturable fungi isolated from this endangered orchid. Endophytic fungi have been reported as mutualists that could show entomopathogenic activity or produce biologically active secondary metabolites (Stone et al., 2000). In orchids, endophytic fungi could exhibit strong antibacterial or antifungal activities as well as plant-growth promoting effect (Chen et al., 2010; Xing et al., 2011). These findings support the notion that endophytic fungi isolated from *S. tigrina* could have potentially diverse applications. On the other hand, it has been shown that epiphytic fungi are determinant for plant health, plant protection (Andrews and Harris, 2000; Santamaría and Bayman, 2005; Rodriguez et al., 2009), and microbial biodiversity maintenance (Huang et al., 2008; Kharwar et al., 2010). However, the relationship between fungal epiphytes and orchids is practically non-existent. In this study, we obtained a higher number of epiphytic isolates than endophytes in all tissues investigated. This behavior has been reported for other plants including *Coffea arabica* leaves and *Eucaluptus citriodora* (Santamaría and Bayman, 2005; Osono, 2008; Kharwar et al., 2010). It would be relevant to examine both the endophytic and epiphytic fungal populations associated with orchids to fully uncover the ecological potential associated to these plants as a valuable source of biotechnological applications.

Current studies of orchid-associated fungi are mainly focused on mycorrhizae (Kristiansen et al., 2001; Yamato et al., 2005; Huang et al., 2014; Oliveira et al., 2014) and endophytic fungi associated with roots and leaves (Chen et al., 2010; Chutima et al., 2011; Tan et al., 2012), leaving aside the study of fungi present in other tissues. In this work, we examined both the endophytic and epiphytic fungal populations in the orchid to fully consider the great microbiological richness associated to this plant. We found that the leaf was the tissue with the greatest diversity of epiphytes, while the root exhibited the highest diversity of endophytic genera, in accordance with the significant microbial interactions that occur in these plant tissues and with previous reports (Vendramin et al., 2010; Sudheep and Sridhar, 2012; Bunch et al., 2013; Chen et al., 2013). However, we also found that other tissues like the pseudobulb showed a considerable diversity for both epiphytic and endophytic isolates, supporting the notion that other tissues should also be considered in this type of studies. Moreover, this capability of S. *tigrina* to relate with many fungal partners could represent a biological and survival advantage for the orchid (Otero et al., 2004).

Concerning genera distribution that conform the epiphytic or endophytic communities isolated from this orchid, we found *Trichoderma* and *Fusarium* as the most prevalent genera for the epiphytic and endophytic communities, respectively. It has been reported that *Trichoderma* produces enzymes involved in cell plant degradation and could be applied for biocontrol (Naher et al., 2014; Schmoll et al., 2016), while *Fusarium* produces secondary metabolites such as gibberellins (O'Donnell et al., 1998; Malonek et al., 2005) and protect plants from pathogenic isolates (Minerdi et al., 2011). Besides these prevalent fungi, we identified 25 genera as part of both the epiphytic and endophytic communities in *S. tigrina*, where the most widely distributed genera were *Paecilomyces, Penicillium*, and *Scedosporium*. These fungal isolates could be applied to benefit the orchid by inducing growth promotion through hormone secretion, by generating plant resistance due to the activation of multiple defense signals or they could also show antagonistic activity against pathogens, as reported for other hosts (Kiewnick and Sikora, 2006; Hossain et al., 2007; Khan et al., 2011a; Radhakrishnan et al., 2013).

Even when several genera showed ubiquitous distribution, some appeared to have a preference. This was the case for *Alternaria, Annulohypoxylon, Colletotrichum*, and *Phaeosphaeriopsis* which were recovered only as part of the endophytic community and their natural ability to inhabit the orchid could be advantageous for future biotechnological applications. For example, *Annulohypoxylon* (previously referred as *Hypoxylon*) presents an endophytic stage as part of its life cycle and produces several secondary metabolites with antibiotic and antiparasitic activities (Piettre et al., 2002; Bills et al., 2012). On the other hand, we found that 31 genera were exclusively epiphytic, which could be related mainly to the general richness observed for this community.

### Fungal gibberellin producers

It has been reported that GA_3_ has the ability to alter orchid seedling morphology by elongating seedlings (Hadley and Harvais, 1968; Ávila-Díaz et al., 2009) and GAs are known to promote cell elongation and division in various plant tissues (Potter et al., 1993; Bianco et al., 1996; Ozga et al., 2002; Kazmierczak, 2003; Little and MacDonald, 2003; Bilkay et al., 2010). Orchid embryos typically form globular structures, expanding outward in all directions and germination could conceivably be enhanced by the action of phytohormones like gibberellins. Here, we identified several gibberellin-producing isolates in suitable yields according to concentrations that have shown biological activity in different plants (Hamayun et al., 2009, 2010b; Khan et al., 2011c; Waqas et al., 2012), suggesting that these isolates could be useful to improve propagation, adaptation, and conservation of *S. tigrina*, particularly at early stageS. When identified, these isolates were associated to 11 genera that comprise *Penicillium* and *Arthrinium*, which have already been reported as gibberellin-producers (Mitter et al., 2002; Kawaide, 2006; Khan et al., 2009; Bahalla et al., 2010; Leitão and Enguita, 2016) with the ability to enhance plant growth and to help the plants to resist abiotic stresses (Hamayun et al., 2010a; Khan et al., 2011b, 2015a).

Gibberellins play a relevant role in plant growth and development. However, many fungi that associate with plants or soil have not been tested for their ability to produce GAs. Here, we found that isolates with the ability to produce gibberellins displayed a high degree of similarity with the following genera, according to the fungal ITS regions: *Bionectria, Diplodia, Macrophoma, Nectria, Neopestalotiopsis, Talaromyces*, and *Trichoderma*. Even when we found a high similarity when identifying the isolates, further investigations will help to fully characterize the identity of these isolates and their ability to produce gibberellins and other useful metabolites. Finally, the physiological effects of this metabolite in orchids, particularly in *S. tigrina* remains to be elucidated.

Overall, this study shows novel information about the composition of the mycobiome present in *S. tigrina*, an endangered species of the rich endemic Mexican ecosystem. As found for other orchids, *S. tigrina* shows a complex fungal community that includes diverse groups, suggesting the probable relevance of its biodiversity in maintaining equilibrium with its environment. Moreover, interesting insights related to the composition of the epiphytic and endophytic communities and their distribution in the plant showed a great fungal richness for this orchid. Finally, we believe that the description of the natural mycobiome associated to *S. tigrina* and the capability of some of the isolates to produce secondary metabolites could be applied in different biotechnological strategies oriented to increase the population of *S. tigrina* in Mexico. This integrated approach could benefit delicate members of ecosystems, particularly for endangered species. Similar studies could be conducted to gain insights into the fungal microbiome associated to other plants, in order to identify microbial symbionts that may play a role in providing stability for the host in their particular ecosystem.

## Author contributions

Overall approach of this study was designed by NM-M and RM-C. SS-C conducted the experiments. MC-L designed and supervised HPLC experiments. All authors were involved in experiment design, data analysis, and manuscript preparation.

### Conflict of interest statement

The authors declare that the research was conducted in the absence of any commercial or financial relationships that could be construed as a potential conflict of interest.
