# Supplementary material for: Fungal Diversity and Community Composition of Culturable Fungi in Stanhopea trigrina Cast Gibberellin Producers
Source: Front Microbiol. 2018 Apr 4;9:612. doi: 10.3389/fmicb.2018.00612 (PMC5893766; doi:10.3389/fmicb.2018.00612)
Supplement: Supplementary file 1 [file Table1.DOCX]

**Table S1** Description of the fungal morphotypes identified

| Genus | Morphotype | Characteristics | N°  Isolates |  |  |
| --- | --- | --- | --- | --- | --- |
| *Acremonium* | 1 | Floccose texture colonies, the color of mycelia are white | 4 | 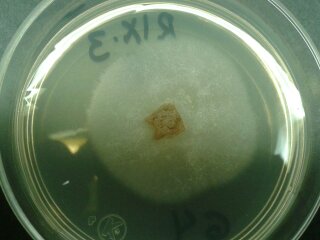 | 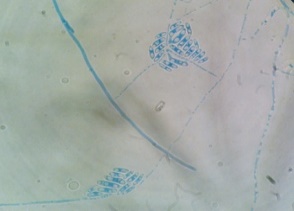 |
|  | 2 | Colonies floccose, in soft cream color | 7 | 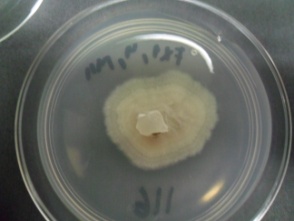 | 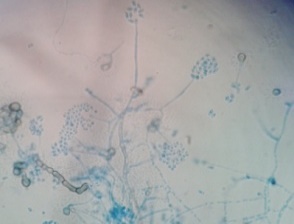 |
|  | 3 | Floccose texture colonies, the color of mycelia are lightly yellow | 5 | 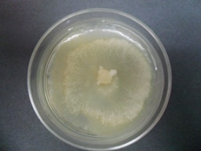 | 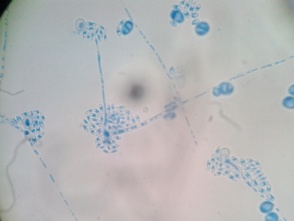 |
|  | 4 | Colonies floccose, in soft pink color | 1 | 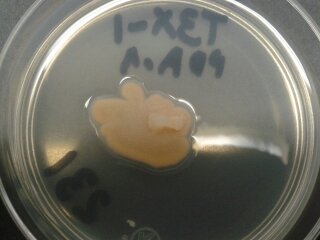 | 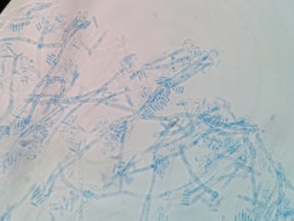 |
| *Alternaria* | 5 | Texture wooly, color pale gray | 1 | 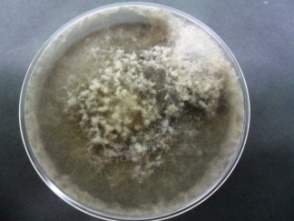 | 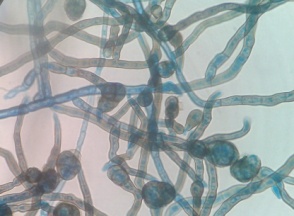 |
| *Annulohypoxylon* | 6 | Filamentous colony of abundant growth, cream color | 2 | 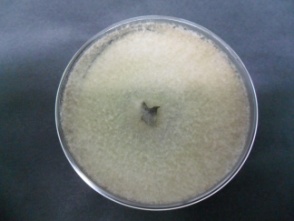 | 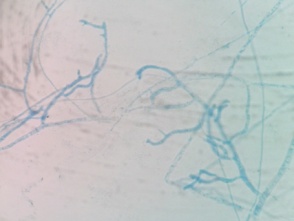 |
| *Anthostomella* | 7 | White filamentous flat colony | 8 | 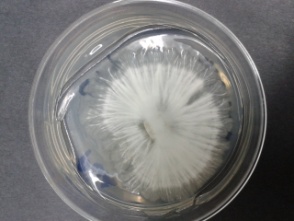 | 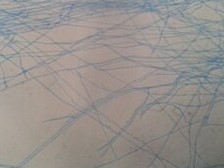 |
| *Aplosporella* | 8 | Colony circular dense, initially greenish, becoming gray | 16 | 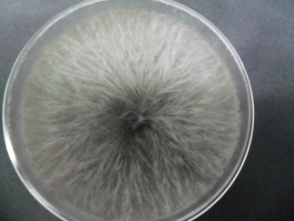 | 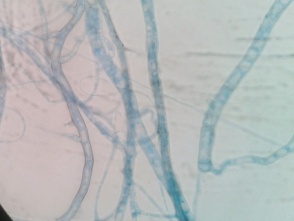 |
| *Arthopyrenia* | 9 | flat pink colony | 1 | 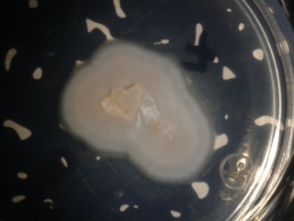 | 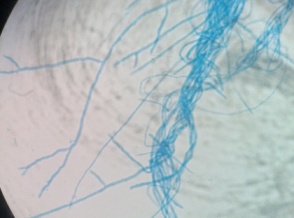 |
|  | 10 | Colonies flat, with sparse aerial mycelium white | 2 | 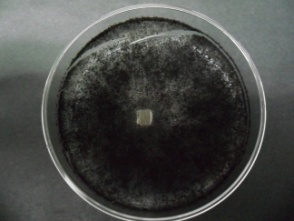 | 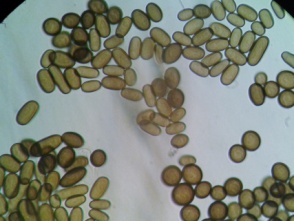 |
| *Arthrinium* | 11 | White filamentous colonies with moderate growth | 8 | 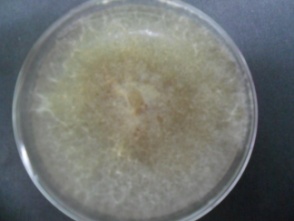 | 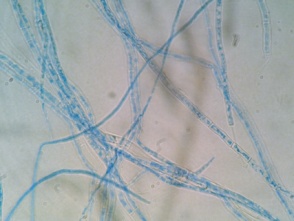 |
|  | 12 | Colonies flat, moderate aerial mycelium, reverse pale luteous | 15 | 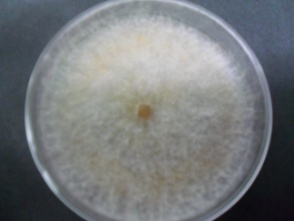 | 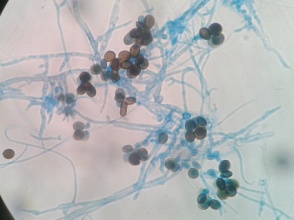 |
|  | 13 | Cottony gray colony | 5 | 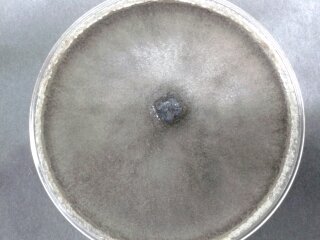 | 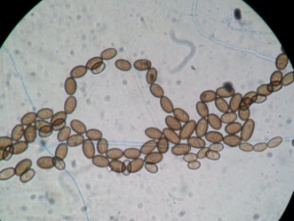 |
| *Aspergillus* | 14 | Colonies velvety and white | 7 | 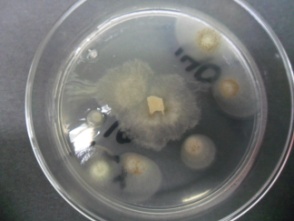 | 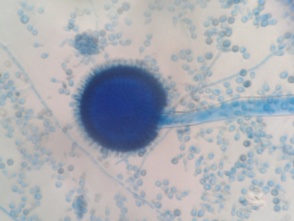 |
|  | 15 | Gray filamentous colony that pigmenting medium in green | 1 | 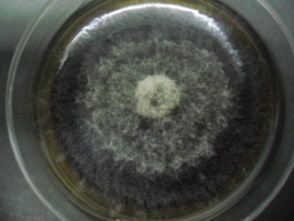 | 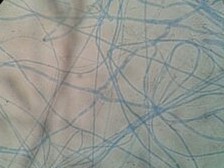 |
|  | 16 | Colonies velvety and white, with brown spots on the surface Colonies powdery, green, reverse is tan | 13 | 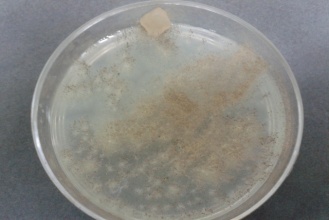 | 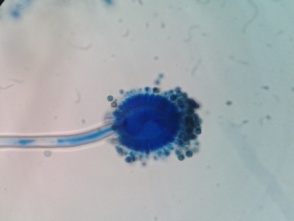 |
|  | 17 | Pale brown velvety colonies | 2 | 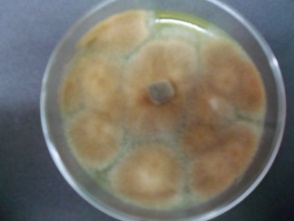 | 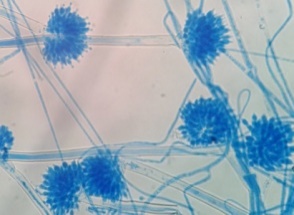 |
|  | 18 | Filamentous light growth in salmon color | 1 | 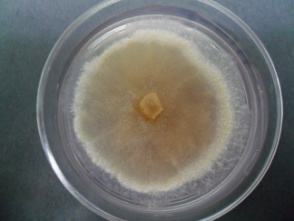 | 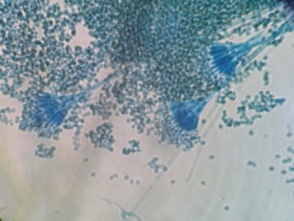 |
|  | 19 | Colonies are velvety, orange-brown with folds | 2 | 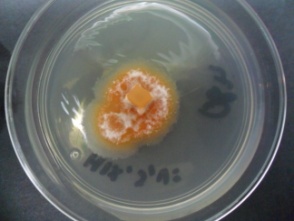 | 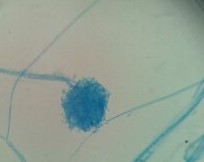 |
|  | 20 | Colonies with smooth Surface in beige color | 6 | 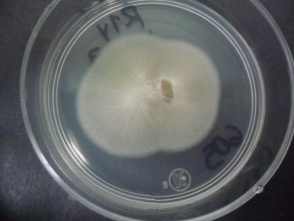 | 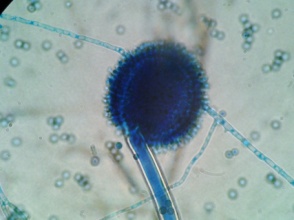 |
| *Bionectria* | 21 | Flat and cotton colony in cream color | 4 | 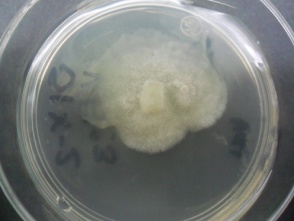 | 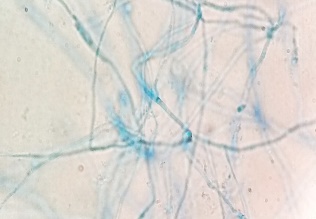 |
| *Bipolaris* | 22 | Limited, filamentous-hairy colony, immersed in agar, brown | 1 | 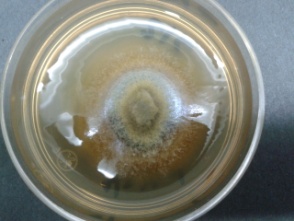 | 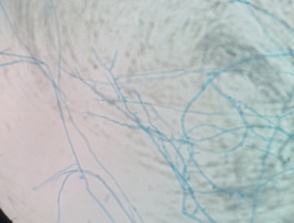 |
|  | 23 | Dark brown filament colony | 1 | 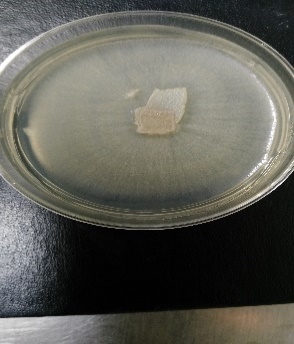 | 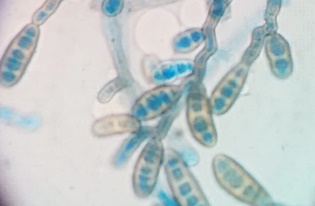 |
| *Chaetomium* | 24 | Filamentous light growth in white color | 2 | 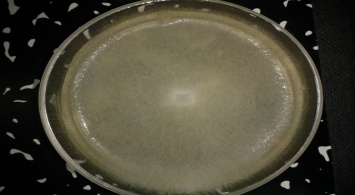 | 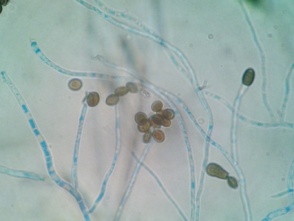 |
|  | 25 | White colonies with cottony mycelium | 3 | 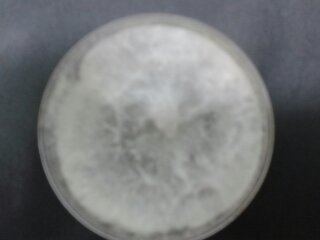 | 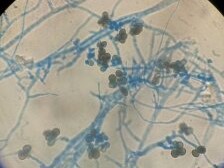 |
| *Chaunopycnis* | 26 | Small white colonies | 10 | 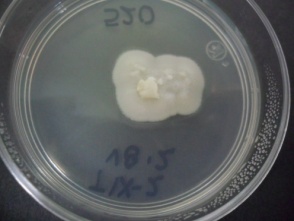 | 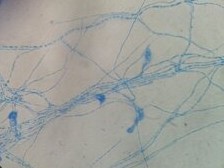 |
| *Cladophialophora* | 27 | Black velvety colonies | 1 | 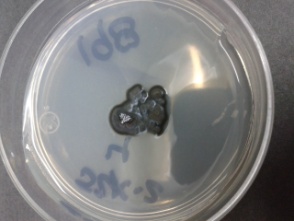 | 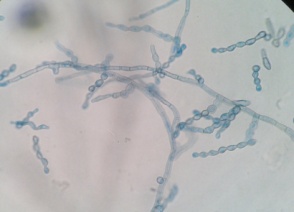 |
| *Cladosporium* | 28 | Velvety colonies in olive green | 5 | 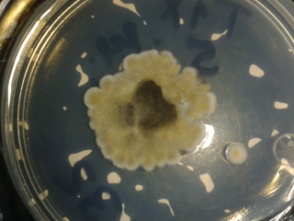 | 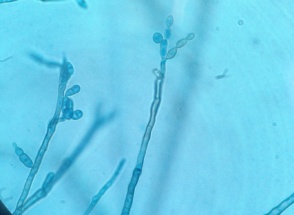 |
|  | 29 | Velvety colonies in dark green | 1 | 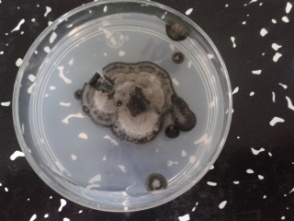 | 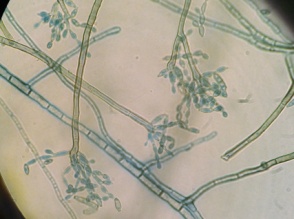 |
| *Cochliobolus* | 30 | Colony limited, white center yellow color, creamy-rough appearance | 1 | 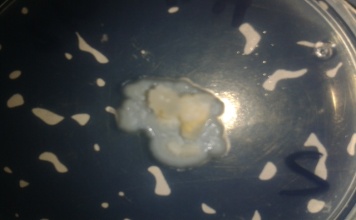 | 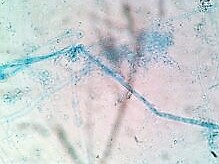 |
| *Colletotrichum* | 31 | Filamentous colony unlimited, grows throughout the plaque, hairy and gray | 1 | 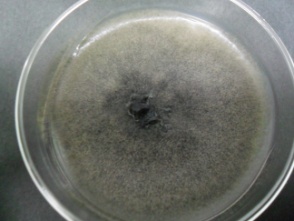 | 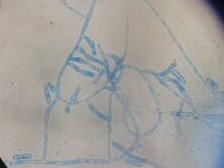 |
| *Curvularia* | 32 | Velvety colonies in dark green | 4 | 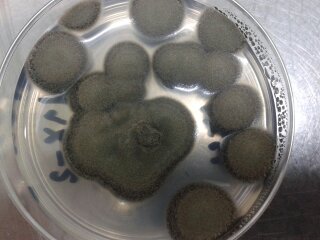 | 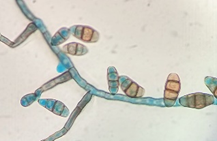 |
| *Daldinia* | 33 | Soft, velvety, limited colony | 14 | 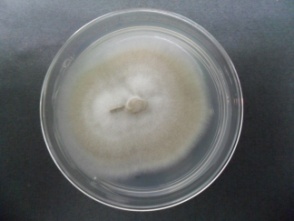 | 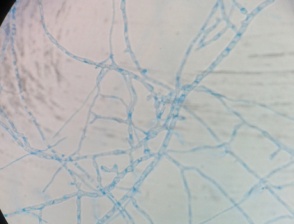 |
|  | 34 | Colony limited, hairy-granular appearance, salmon color | 1 | 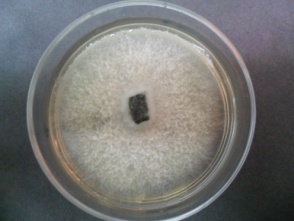 | 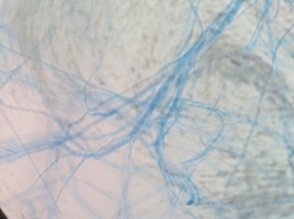 |
| *Diaporthe* | 35 | Filamentous granular colony, white with the reverse light brown | 4 | 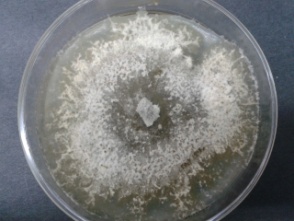 | 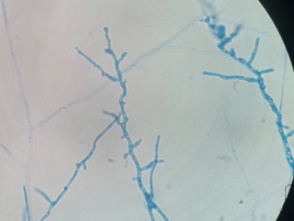 |
| *Diplodia* | 36 | Colony limited, hairy appearance and white color | 4 | 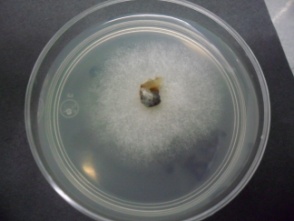 | 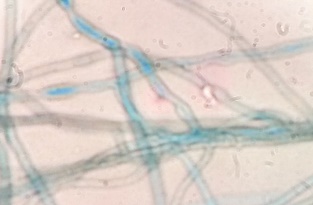 |
| *Elaphocordyceps* | 37 | White cottony colony | 1 | 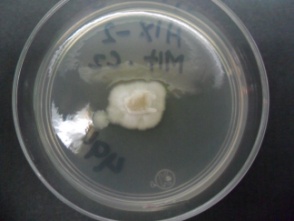 | 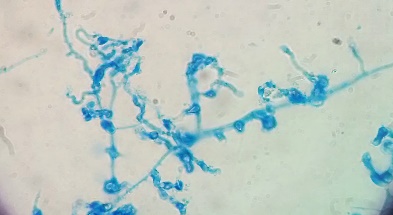 |
| *Epichloe* | 38 | Colony limited, creamy, convex, hard in white | 1 | 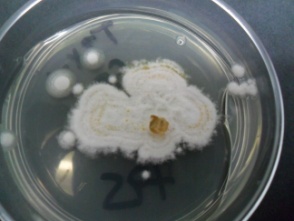 | 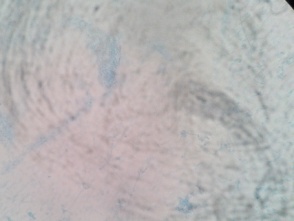 |
|  | 39 | White, cottony cologne, pigments in medium in tan | 2 | 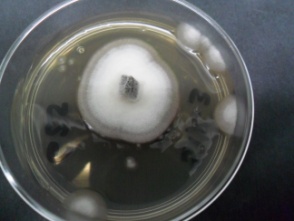 | 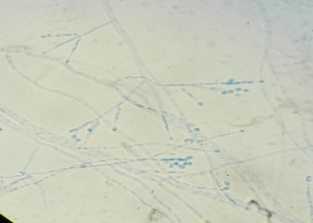 |
| *Eutypa* | 40 | White light radial growth colony | 1 | 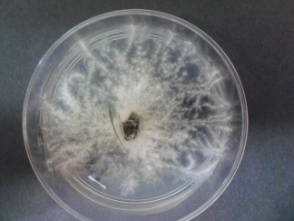 | 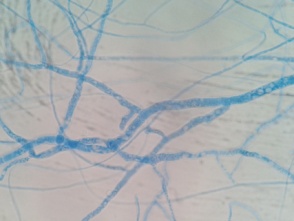 |
| *Eutypella* | 41 | Unlimited cologne, white color, radial | 4 | 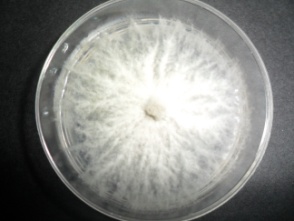 | 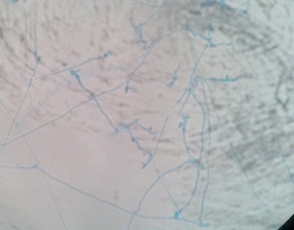 |
| *Exophiala* | 42 | Colony limited, hairy, green color | 1 | 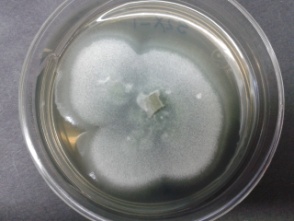 | 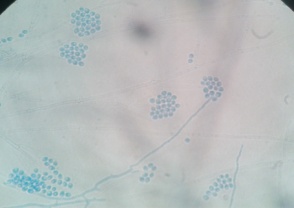 |
|  | 43 | Colony limited, creamy, convex, smooth olive green | 1 | 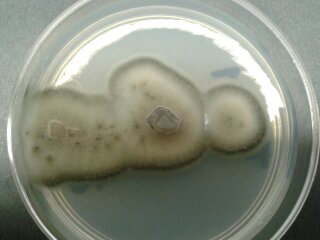 | 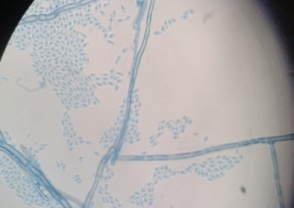 |
| *Fonsecae* | 44 | Velvety green cologne, limited | 11 | 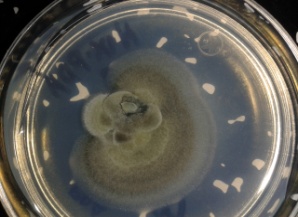 | 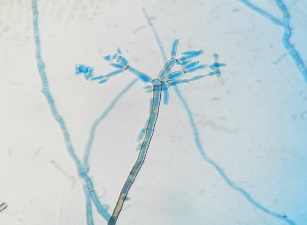 |
| *Fusarium* | 45 | Cologne unlimited size, white, hairy-dry appearance | 24 | 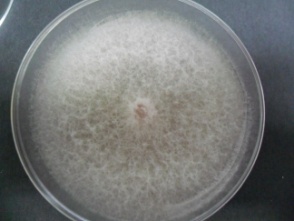 | 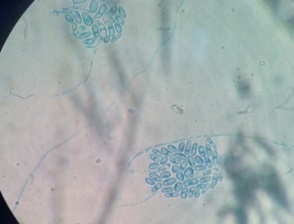 |
|  | 46 | Unlimited cologne, of appearance; hairy, orange-carrot | 2 | 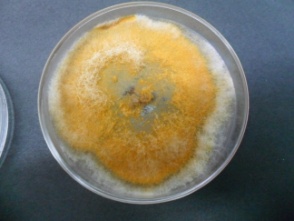8 | 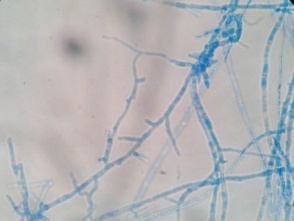 |
|  | 47 | Unlimited cologne, hairy, flat, white | 8 |  | 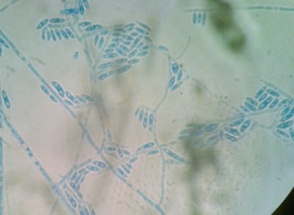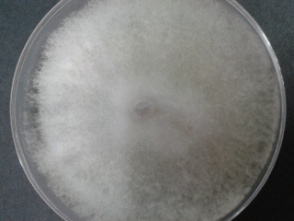 |
|  | 48 | Cologne unlimited hairy look-gray granuler gray | 2 | 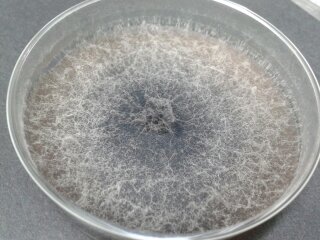 | 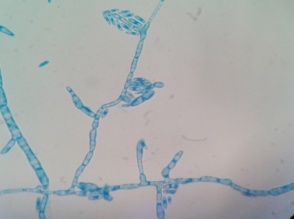 |
|  | 49 | Unlimited colony, hairy red in the center, white periphery, no pigmentation in the middle | 2 | 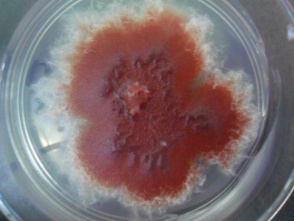 | 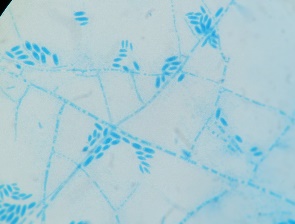 |
|  | 50 | Limited colony of hairy appearance, white color with bulging center, gray coloration in the periphery | 2 | 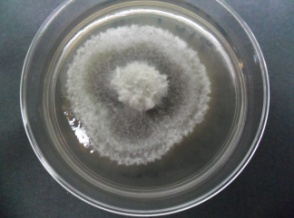 | 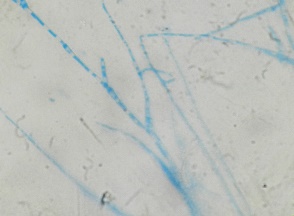 |
| *Fusicolla* | 51 | Cologne limited, hard, convex, smooth orange-brown | 1 |  |  |
| *Geotrichum* | 52 | Colony limited, white color, hairy, flat with bulky center | 5 |  |  |
|  | 53 | Unlimited cologne, of appearance; hairy-dry, white | 9 |  |  |
| *Gibberella* | 54 | Colony limited, hairy white | 1 |  |  |
| *Hypocrea* | 55 | Colony limited, hairy white | 1 |  |  |
| *Hypoxylon* | 56 | White cottony colony with the reverse brown | 6 |  |  |
| *Letendraea* | 57 | White cottony colony, bulging center | 1 |  |  |
| *Macrophoma* | 58 | Unlimited cologne, hairy-granular appearance, in coffee color, with pigmentation grape in the middle | 2 |  |  |
|  | 59 | Irregular cologne with cottony-fluffy appearance in gray | 4 |  |  |
| *Microdiplodia* | 60 | Gray color; hairy appearance, pigmentation medium brown | 1 |  |  |
|  | 61 | Cologne unlimited, hairy-granular appearance in brown-yellow color | 2 |  |  |
| *Microsporum* | 62 | Colony size limited, white; hairy appearance, bulging center | 2 |  |  |
| *Mucor* | 63 | Filamentous colony, thin hairy-filament look in white | 8 |  |  |
|  | 64 | Unlimited cologne; appearance: fluffy, yellow | 13 |  |  |
|  | 65 | Colony limited, dark green, velvety, rough | 1 |  |  |
|  | 66 | Unlimited cologne, hairy, flat, white | 5 |  |  |
| *Nectria* | 67 | White cottony colony, with medium pigment in coffee | 1 |  |  |
|  | 68 | Unlimited cologne white, no pigmentation in the middle | 4 |  |  |
| *Neopestalotiopsis* | 69 | Unlimited cologne white, no pigmentation in the middle | 9 |  |  |
| *Nigrograna* | 70 | Cotton cologne color brown tenuous | 1 |  |  |
| *Ochlorocladosporium* | 71 | Flat, velvety and olive green colony | 2 |  |  |
| *Paecilomyces* | 72 | Dark green colonies with suede-like texture | 1 |  |  |
|  | 73 | Colonies powdery, green, reverse is tan | 1 |  |  |
|  | 74 | Flat colonies with suede-like texture in peach color | 1 |  |  |
|  | 75 | Colony powdery olive green | 4 |  |  |
|  | 76 | Rugose in the center, filamentous around. Pigment the medium in yellow | 1 |  |  |
|  | 77 | Granular flat colony in light pink color | 4 |  |  |
|  | 78 | cotton colony color pale pink | 1 |  |  |
|  | 79 | Filamentous circular colony in soft yellow | 3 |  |  |
| *Paraconiothyrium* | 80 | Limited, hairless, brown cologne | 4 |  |  |
|  | 81 | Filamentous brown colony, growing thin | 1 |  |  |
|  | 82 | Colony flat, velvety in brown with green | 5 |  |  |
|  | 83 | Colony circular, velvety in cream color | 6 |  |  |
|  | 84 | Flat, filamentous colony, light yellow, pigmented the medium in light pink | 2 |  |  |
|  | 85 | Delicate filamentous colony with orange color on the periphery | 1 |  |  |
|  | 86 | Flat colony with delicate filamentous growth in peach color | 23 |  |  |
|  | 87 | Limited colony, bulging hairy appearance in white | 9 |  |  |
|  | 88 | Texture velvety to powdery; green | 10 |  |  |
| *Penicillium* | 89 | Flat, velvety and orange cologne | 1 |  |  |
|  | 90 | Whitish yellow filamentous colony | 4 |  |  |
|  | 91 | Irregular colony rough in white color | 3 |  |  |
|  | 92 | Filamentous colony with abundant growth in gray color | 2 |  |  |
|  | 93 | Filaments cream color colony | 10 |  |  |
|  | 94 | Circular cotton colony light brown color | 3 |  |  |
|  | 95 | Flat colony, velvety in green | 1 |  |  |
|  | 96 | Filamentous colony in light brown | 13 |  |  |
|  | 97 | Filamentous colony cream color with pink center | 1 |  |  |
|  | 98 | Cotton-white cologne | 7 |  |  |
|  | 99 | Flat, filamentous colony, light brown in the center, pigmented the medium in red | 1 |  |  |
| *Pestalotiopsis* | 100 | Yellow, velvety-granular, flat colony, limited | 9 |  |  |
|  | 101 | Colony limited, irregular hairy brown, filaments submerged in the center | 1 |  |  |
| *Phaeosphaeriopsis* | 102 | Cologne, hairy, limited coffee-cream, concentric, flat | 1 |  |  |
| *Phialemoniopsis* | 103 | Colony limited, creamy, hard appearance | 2 |  |  |
|  | 104 | Colony limited, cottony-looking center bulging in gray, and white on the periphery | 2 |  |  |
| *Phoma* | 105 | Colony limited, hairy in yellow pigmentation to the medium thin coffee | 1 |  |  |
| *Pseudobotrytis* | 106 | Colony limited, creamy, hard and rough in green color, obverse grape | 1 |  |  |
| *Pyrenochaeta* | 107 | Unlimited cologne, delicate hairy, flat, with gray sporulation | 2 |  |  |
| *Pyrenochaetopsis* | 108 | Colonia dura, cremosa, convexa en color blanco | 2 |  |  |
| *Roussoella* | 109 | cotton-colored cologne cream center slightly bulging | 2 |  |  |
|  | 110 | Unlimited, irregular, powdery-looking cologne in black | 1 |  |  |
| *Scedosporium* | 111 | Colony limited, convex and plagued in brown | 3 |  |  |
|  | 112 | Gray-powdery colony, limited, flat, gray | 1 |  |  |
|  | 113 | Colony limited, filamentous-hairy, white | 7 |  |  |
|  | 114 | Colony limited, hairy appearance, white | 3 |  |  |
|  | 115 | Colony limited, gray hairy | 1 |  |  |
|  | 116 | Colony limited, filamentous-creamy appearance in purple | 1 |  |  |
|  | 117 | Colony limited, coffee creamy center, delicate filament in the periphery | 1 |  |  |
| *Schizophyllum* | 118 | Limited, looking cologne; fluffy-dry, creamy | 1 |  |  |
| *Talaromyces* | 119 | Colony unlimited, hairy-cottony orange | 1 |  |  |
| *Thyridaria* | 120 | Creamy cotton cologne | 1 |  |  |
| *Trichoderma* | 121 | Unlimited colony of white color and presents granulations in the center, no pigmentation is observed in the medium | 14 |  |  |
|  | 122 | Unlimited cologne, hairy, flat, white, with sporulation in green color | 62 |  |  |
|  | 123 | Cologne limited, velvety concentric appearance in light brown | 1 |  |  |
|  | 124 | Limited colony, hairy-filamentous in white, no pigmentation in the medium | 2 |  |  |
|  | 125 | Unlimited cologne, fill the plate. Hairy and white. | 28 |  |  |
|  | 126 | Filamentous colony, flat, delicate yellow. | 6 |  |  |
|  | 127 | Unlimited colony, filamentous-hairy, white in color with red center | 3 |  |  |
|  | 128 | Unlimited cologne, fill plate; of appearance and form: hairy in white color | 9 |  |  |
| *Trichophyton* | 129 | Creamy, limited colony, convex and flat | 2 |  |  |
| *Umbelopsis* | 130 | Filamentous cologne salmon color, pigments to the medium in color tenuous grape | 2 |  |  |
| *Verticillium* | 131 | Colony limited, radial, white color, hairy-cottony appearance | 13 |  |  |
| *Xylariaceae* | 132 | Colony limited in white color | 1 |  |  |
|  | 133 | Colony limited, hairy-granular in white color | 1 |  |  |
| *Xylariales* | 134 | Colony limited, fluffy, center bulging in white | 9 |  |  |
| Unidentified | 135 | White-yellow color, hairy-cottony appearance | 1 |  |  |
| Unidentified | 136 | Filamentous colony, flat, white, center coffee like yeast. | 1 |  |  |
| Unidentified | 137 | Unlimited colony, hairy-flat, white | 3 |  |  |
| Unidentified | 138 | Limited, irregular, granular colony in yellow color, gelatinous consistency | 2 |  |  |
| Unidentified | 139 | Limited cologne, white cottony | 3 |  |  |
