# Supplementary figures and images for: Fungal Diversity and Community Composition of Culturable Fungi in Stanhopea trigrina Cast Gibberellin Producers
Source: Front Microbiol. 2018 Apr 4;9:612. doi: 10.3389/fmicb.2018.00612 (PMC5893766; doi:10.3389/fmicb.2018.00612)

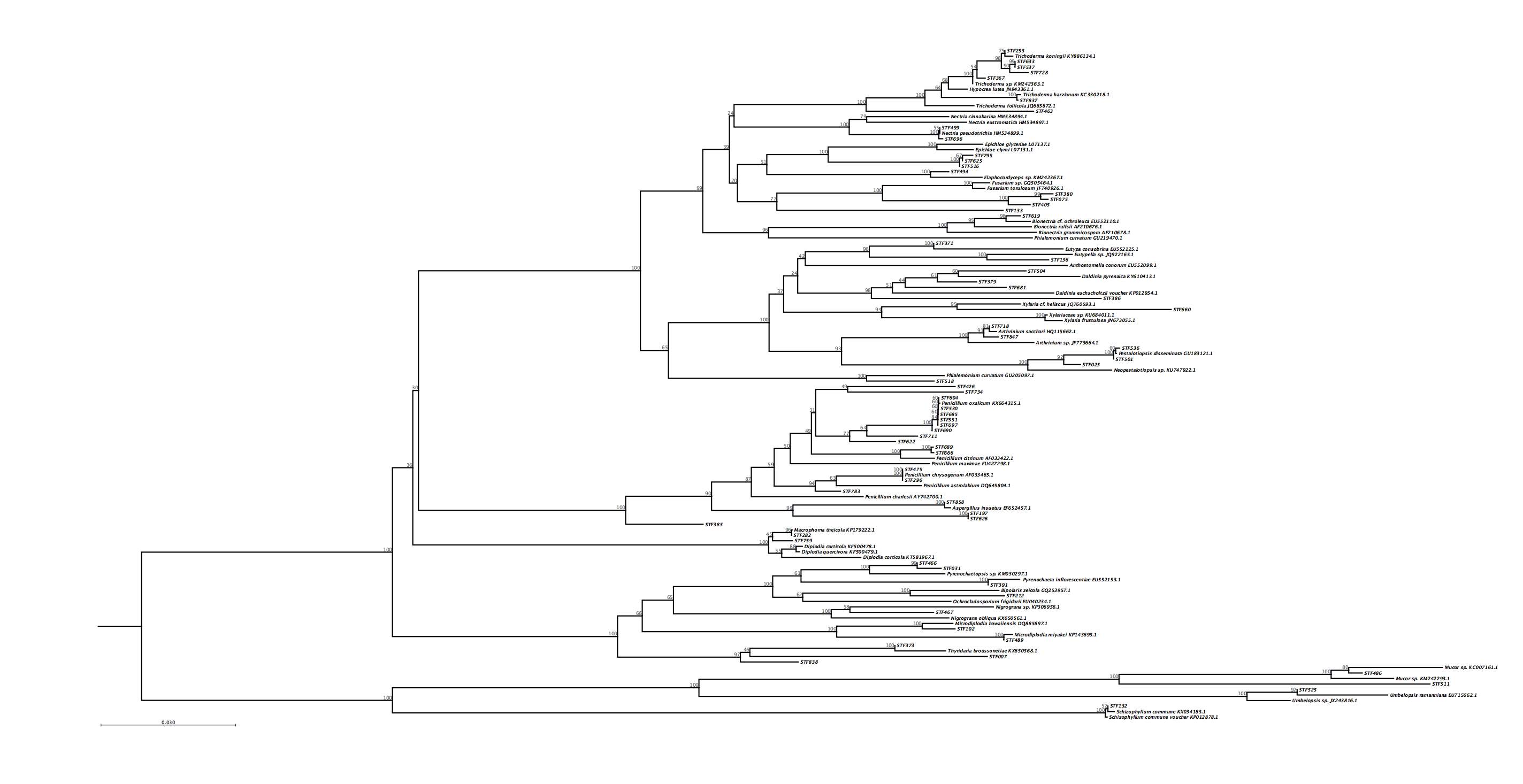

Supplement: Supplementary file 3 [file Image1.TIFF]
