# Supplementary material for: Fungal Diversity and Community Composition of Culturable Fungi in Stanhopea trigrina Cast Gibberellin Producers
Source: Front Microbiol. 2018 Apr 4;9:612. doi: 10.3389/fmicb.2018.00612 (PMC5893766; doi:10.3389/fmicb.2018.00612)

**Figure S2. Gibberelin production in fungal isolates determined by HPLC.** The HPLC chromatograms are presented for the standard GA_3_ (1 μg mL−1), the standard GA_4_ (1 μg mL−1) and the different isolates that produce gibberellins. The ID for the different isolates is included for each chromatogram.


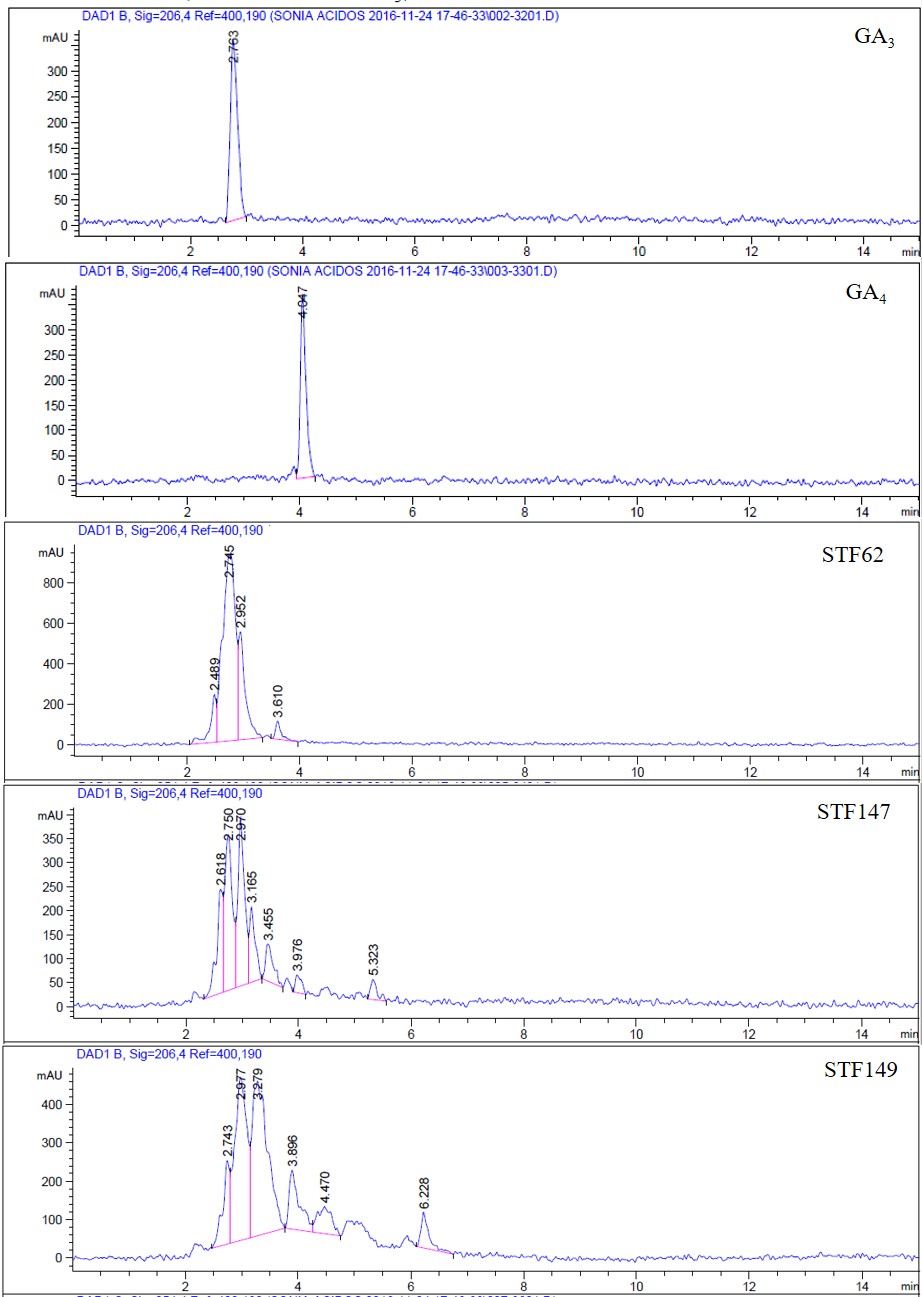


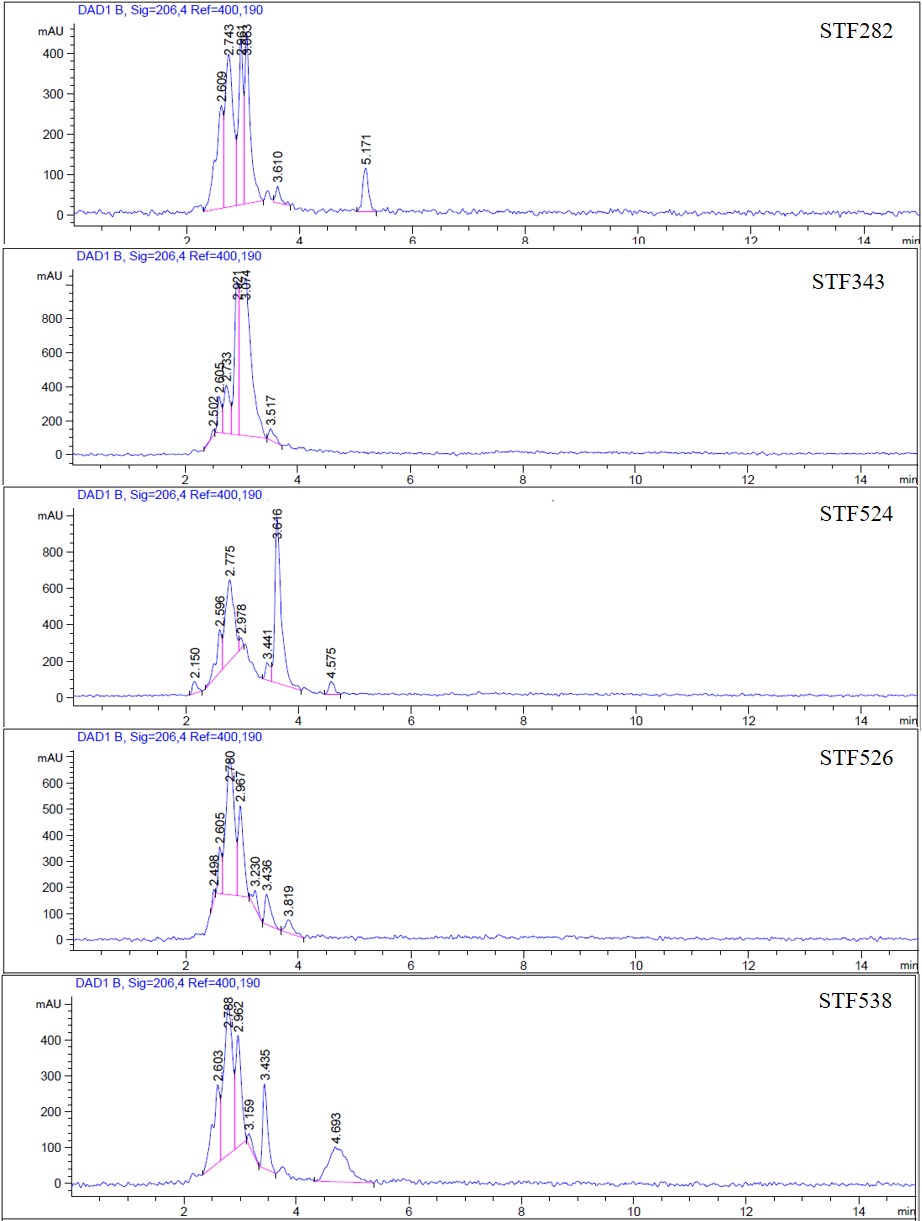


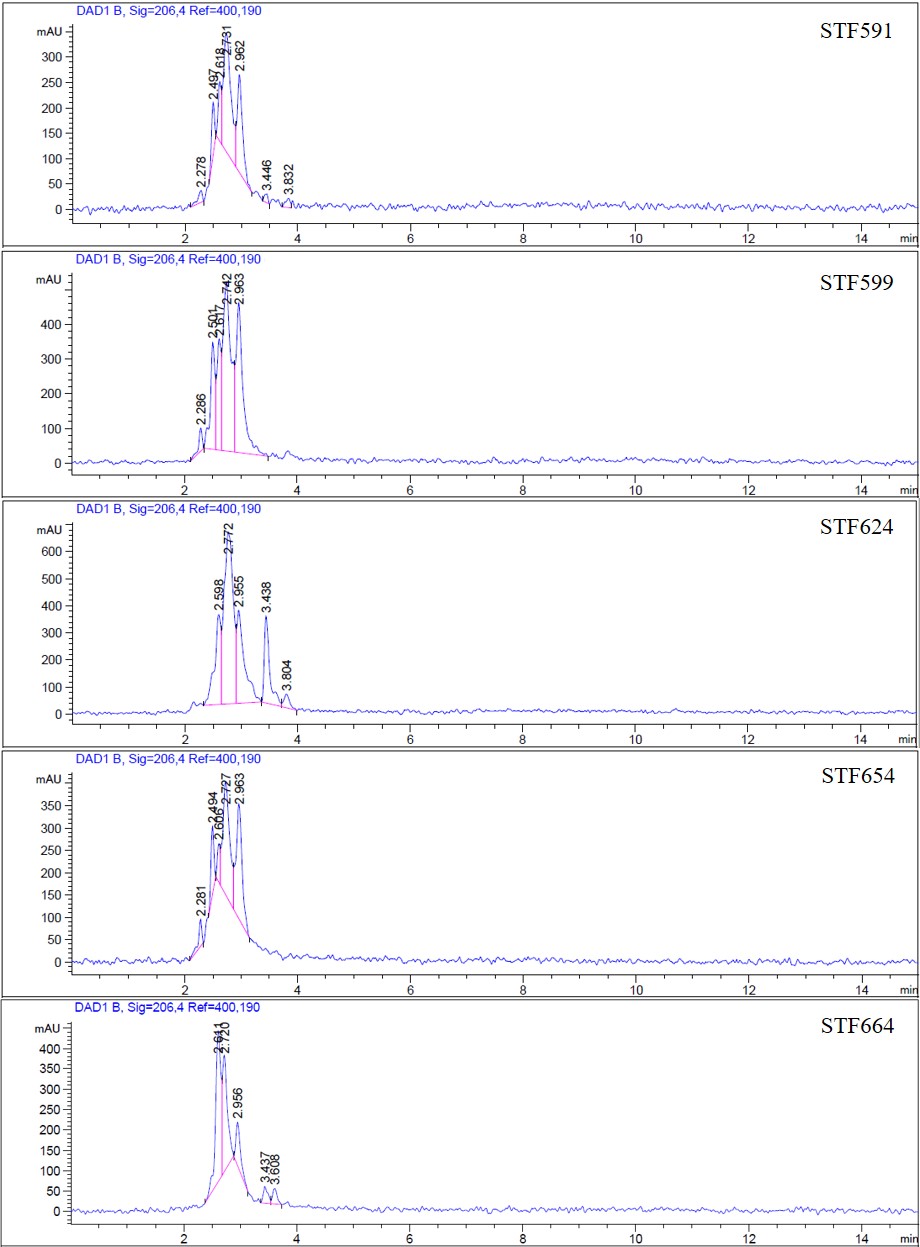


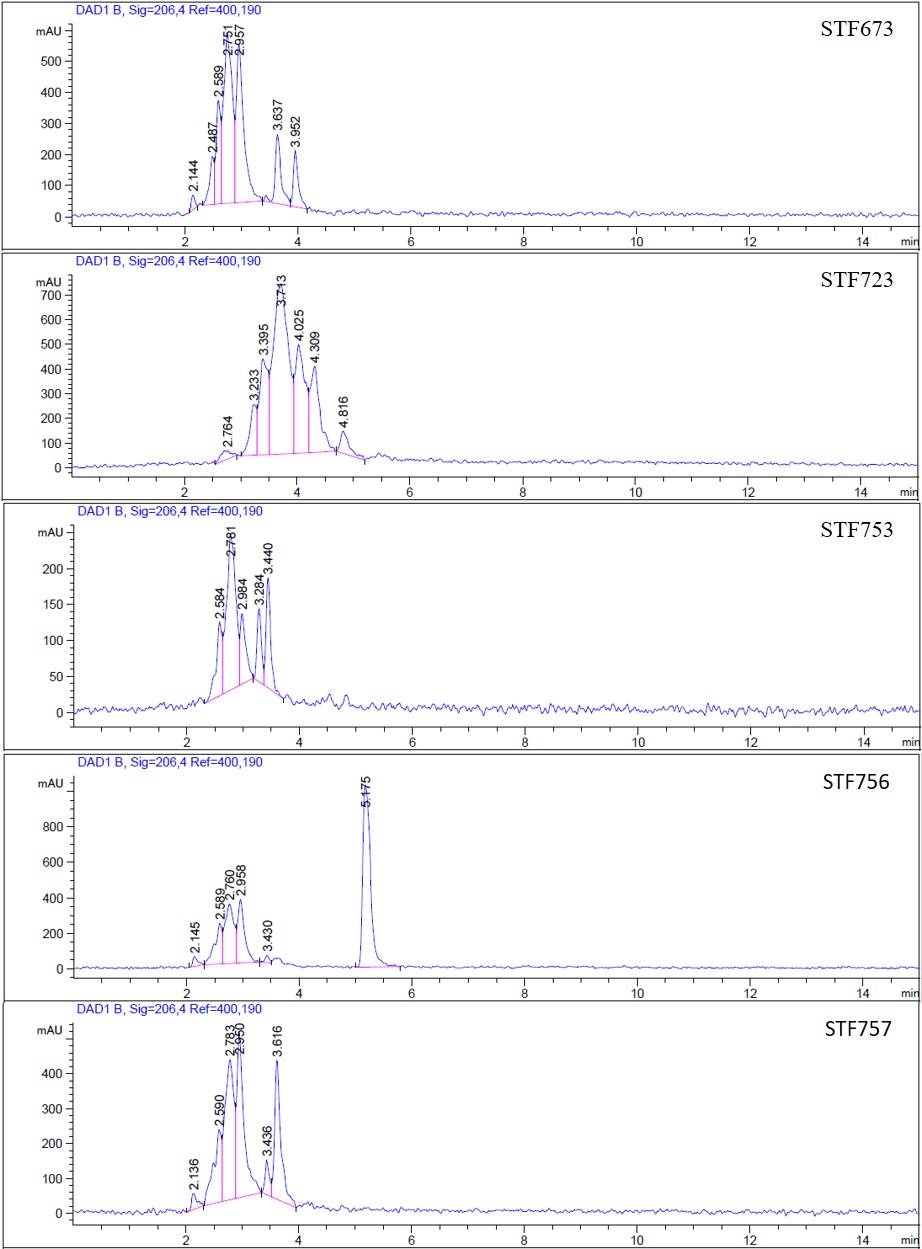


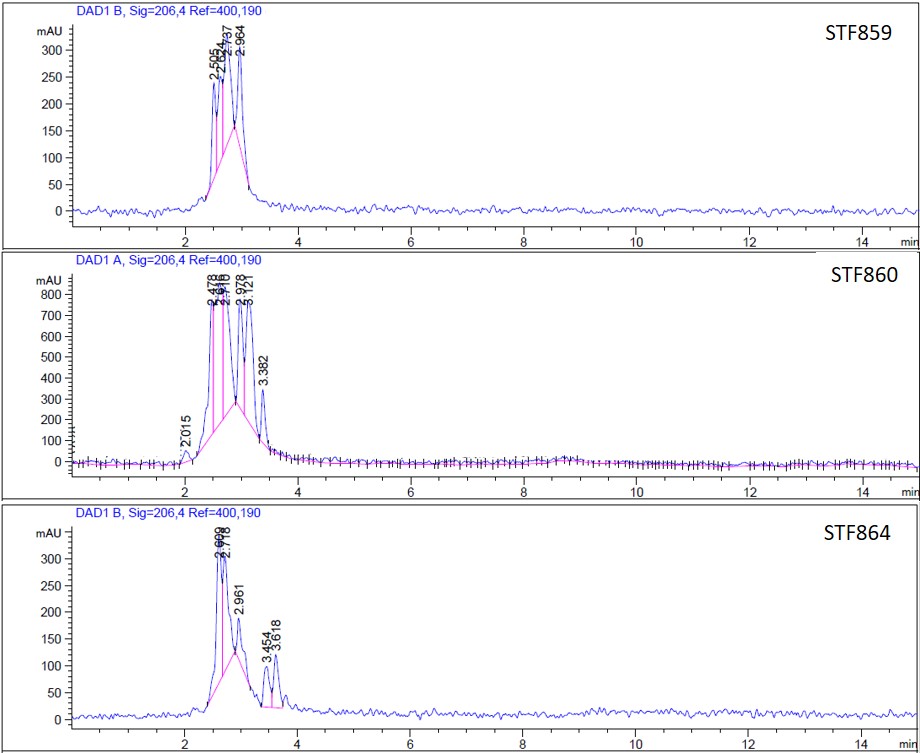

Supplement: Supplementary file 4 [file DataSheet1.docx]
